# Optimisation of a TALE nuclease targeting the HIV co-receptor CCR5 for clinical application

**DOI:** 10.1038/s41434-021-00271-9

**Published:** 2021-06-11

**Authors:** Lea Isabell Schwarze, Dawid Głów, Tanja Sonntag, Almut Uhde, Boris Fehse

**Affiliations:** 1 Department of Stem Cell Transplantation, Research Department Cell and Gene Therapy, University Medical Centre Hamburg-Eppendorf, Hamburg, Germany; 2 German Centre for Infection Research (DZIF), partner site Hamburg, Hamburg, Germany

**Keywords:** Infectious diseases, Transfection

## Abstract

Disruption of the *C-C-Chemokine-receptor-5* (*CCR5*) gene induces resistance towards CCR5-tropic HIV. Here we optimised our previously described CCR5-Uco-TALEN and its delivery by mRNA electroporation. The novel variant, CCR5-Uco-hetTALEN features an obligatory heterodimeric Fok1-cleavage domain, which resulted in complete abrogation of off-target activity at previously found homodimeric as well as 7/8 in silico predicted, potential heterodimeric off-target sites, the only exception being highly homologous *CCR2*. Prevailing 18- and 10-bp deletions at the on-target site revealed microhomology-mediated end-joining as a major repair pathway. Notably, the CCR5^Δ55–60^ protein resulting from the 18-bp deletion was almost completely retained in the cytosol. Simultaneous cutting at *CCR5* and *CCR2* induced rearrangements, mainly 15-kb deletions between the cut sites, in up to 2% of T cells underlining the necessity to restrict TALEN expression. We optimised in vitro mRNA production and showed that *CCR5*-on- and *CCR2* off-target activities of CCR5-Uco-hetTALEN were limited to the first 72 and 24–48 h post-mRNA electroporation, respectively. Using single-cell HRMCA, we discovered high rates of TALEN-induced biallelic gene editing of *CCR5*, which translated in large numbers of CCR5-negative cells resistant to HIVenv-pseudotyped lentiviral vectors. We conclude that CCR5-Uco-hetTALEN transfected by mRNA electroporation facilitates specific, high-efficiency *CCR5* gene-editing (30%–56%) and it is highly suited for clinical translation subject to further characterisation of off-target effects.

## Introduction

Since the first description of AIDS and its causative agent HIV more than 40 years ago [1, 2], incredible progress in the development of new drugs and therapies has been accomplished. In fact, introduction of anti-retroviral therapy (ART) turned HIV infections into a treatable chronic disease [3, 4]. However, according to UNAIDS out of estimated 38 Mio people infected with HIV worldwide, only 25.4 Mio did have access to ART in 2019. Resultantly, 690 thousand people died from AIDS-related illnesses in 2019.

HIV primarily infects CD4^+^ T cells [5, 6] causing a decline of their numbers and diversity, which ultimately leads to a dysfunctional immune system and AIDS development. Very early during primary infection, subpopulations of HIV-infected, resting memory CD4^+^ T cells become a lifelong HIV reservoir that cannot be affected by ART due to transcriptional silencing of the provirus [7]. Thus, therapy interruption usually leads to rebound of viremia and recovery of systemic infection [8, 9]. Moreover, despite keeping HIV below detectable levels, ART does not fully prevent chronic immune activation, inflammation and slow lymphoid tissue damage [10–12]. Other disadvantages of lifelong ART include evolvement of drug resistance, high costs, decreasing compliance/adherence and typical problems for the majority of continuous therapies—drug toxicity or adverse drug interactions [13]. Altogether, there is a strong need to develop alternative HIV treatments aiming at cure or permanent virus control, which could be referred to as long-term remission.

Especially two reports on long-term HIV remission without ART application have attracted worldwide attention [14, 15]. In both cases, HIV-positive individuals underwent stem cell transplantation from HLA-matched, CCR5-negative donors to treat their malignant disorders. It is well known that a naturally occurring homozygous 32-base-pair (bp) deletion in the open reading frame of the *CCR5* gene (CCR5Δ32) leads to the expression of a non-functional protein. Since in the vast majority of cases initial HIV infection is mediated by CCR5-tropic HIV strains, CCR5Δ32 homozygous individuals are almost completely protected from HIV [16]. Moreover, even the twofold lower expression of the CCR5 receptor in heterozygous individuals leads to less efficient HIV infection [16, 17]. In light of those findings, CCR5 has become a very popular target for HIV therapy.

In our previous study, we showed that a highly active TAL effector nuclease, CCR5-Uco-TALEN, mediated efficient *CCR5* knockout in primary CD4^+^ T cells after ex vivo mRNA electroporation [18]. We also proved that gene-edited T cells were protected from infection with CCR5-tropic lentiviral vectors and replication-competent CCR5-tropic HIV-1_BaL_ strains [18, 19].

In this work, we introduce and thoroughly characterise an improved CCR5-Uco-hetTALEN with an obligatory heterodimeric Fok1-cleavage domain that significantly reduces the probability of off-target activity. In addition, we present protocols for optimised in vitro production of CCR5-Uco-hetTALEN mRNA and for single-cell high-resolution melting-curve analysis (scHRMCA). We show that editing of the *CCR5* gene results in abrogation of CCR5 expression and a loss of susceptibility towards CCR5-tropic HIVenv-pseudotyped vectors. Together with large-scale data [20], these results confirm the high potential of CCR5-Uco-hetTALEN for clinical translation.

## Material and methods

### Single-cell high-resolution melting-curve analysis (scHRMCA)

We used the FACSAria III Cell Sorter (BD Biosciences, San Jose, CA) to sort single cells treated with CCR5-Uco-hetTALEN (or non-treated cells as control) into a 96-well PCR plate containing 10 µl of lysis buffer (50 mM Tris base pH 8.0, 10 mM EDTA, 100 mM NaCl, 0.1 µl proteinase K (QIAGEN, Hilden, Germany)). The plate was incubated at 37 °C for 1 h, and at 95 °C for 10 min [21]. First, PCR was performed using the following primers: nesPCR fw and nesPCR rv (Table S9). 15 µl of PCR mix (ThermoFisher Scientific, Waltham, MA) containing 2.5 µl DreamTaq buffer, 2.5 U DreamTaq, 5 µM primers, 10 µM MgCl_2_, 5 µM dNTPs were added to the lysed cells. The following PCR programme was carried out: 95 °C for 3 min, 25x [94 °C for 30 s, 57 °C for 30 s, 72 °C for 30 s], 72 °C for 3 min. The resulting PCR product was diluted 1:80 with dH_2_O and used as a template for melting-curve analysis at the LightCycler 480 (LC480) Instrument II (Roche Diagnostics, Mannheim, Germany) with primers HRMfw and HRMrv. Twenty microlitres PCR mix: 6 µM of each primer, 50 mM MgCl_2_, 10-µl LC480 High-Resolution Melting Master (Roche Diagnostics, Basel, Switzerland) and 1.5-µl template. HRMCA programme: 95 °C for 10 min, 40x [95 °C 15 s, 60 °C 30 s ramp rate 2.2 °C/s, 72 °C 30 s single acquisition], 72 °C for 3 min, 40 °C for 10 s ramp rate 2.2 °C/s, 65 °C 10 s, 95 °C ramp rate 0.06 °C/s with continuous acquisition, 40 °C 1 s ramp rate 2.2 °C. Non-edited single cells were used as control to compare melting profiles.

### Next-generation amplicon sequencing (amplicon NGS)

Potential off-targets were identified in silico using the online bioinformatics tools PROGNOS [22] and the Paired Target Finder from TAL Effector Nucleotide Targeter 2.0 [23]. Applied search parameters are summarised in Tables S1 and S2. Results from the two online tools (Tables S3 and S4) were compared, and TOP10 loci were selected based on rank, occurrence, genomic location and relevance in haematopoietic cells (Tables S5 and S6). Amplicon NGS was performed for the selected TOP10 possible off-target loci, as well as the on-target *CCR5* using the Illumina MiSeq/NextSeq Platform (Nextara-two-step-protocol, lllumina MiSeq run 2*250 v2) from Illumina Inc. (San Diego, CA). All steps including library preparation, sequencing and bioinformatic analysis were carried out by Microsynth (Balgach, Switzerland). Samples used in both runs are specified in Table S7. All reads were demultiplexed, trimmed, paired, aligned to the reference genome and analysed for insertions and deletions (Indels). All reads containing insertions and deletions at and/or between TALEN binding sites (‘target region’) were considered editing-induced. Reads without Indels at the target region site were counted as wildtype (WT). Statistical analysis of Indel ratios was done using a one-tailed Welch’s *t*-test. Graphs showing Indel distributions for *CCR5* and *CCR2* were computed using the web-based tool CRISPRESSO 2 (Version 2.0.40) [24]. All other graphs, as well as statistical analyses were performed using GraphPad Prism 8.4.3.

### Cloning of CCR5-Uco-TALEN with codon-optimised Fok1 variants

Heterodimeric Fok1 variants were designed according to Doyon et al. [25] for cleavage domains ELD, KKR, DAD and RVR (Table S8). All used Fok1 variants were codon-optimised using the GeneOptimizer technology and ordered via GeneArt Gene Synthesis (ThermoFisher, Germany). Gene fragments were cloned into vectors pCCR5-Uco-TALEN L (‘left’) and pCCR5-Uco-TALEN R (‘right’) using restriction enzymes *Ear*I (ThermoFisher Scientific) and *Xho*I (ThermoFisher). Each Fok1 variant was cloned into both left and right TALEN vectors.

### Cloning of mRNA production plasmid

For large-scale mRNA production, a 120-nt long poly(A)-sequence was cloned behind the open reading frame of CCR5-Uco-hetTALEN into pCCR5-Uco-hetTALEN(ELD) L and pCCR5-Uco-hetTALEN(KKR) R vectors together with a *Sap*I restriction site for vector linearization. In addition, the AmpR ampicillin resistance gene in the plasmids pCCR5-Uco-hetTALEN(ELD)_poly(A) L and pCCR5-Uco-hetTALEN(KKR)_poly(A) R was replaced by Neomycin phosphotransferase II [NPT II] conferring resistance to kanamycin.

### Cloning of CCR5^Δ55-60^ containing vector

The 18-bp deletion most abundant in *CCR5*-edited T cells results in a 6-amino-acid deletion in the first intracellular loop (ICL1) of CCR5. To study the expression and properties of the resulting CCR5^Δ55-60^ protein, the 18-bp deletion was introduced in *CCR5* by site-directed mutagenesis with primers Rccr5del6fw and Fccr5del6fw (Table S9) performed on plasmid LeGO-CCR5-iB2-Puro+ [19, 26].

### mRNA production

CCR5-Uco-hetTALEN L + R mRNAs were obtained from BioNTech IMFS (Idar-Oberstein, Germany) or produced in our own lab. In-house manufacture was performed by in vitro transcription from T7-plasmids containing CCR5-Uco-TALEN L and R or CCR5-Uco-hetTALEN L and R using the T7 mScript Standard mRNA Production System (CELLSCRIPT, Madison, WI) following the manufacturer’s protocol for RNA production including DNase I treatment and addition of a cap-1 structure. When the poly(A)-tail was not part of the production plasmid, polyadenylation was performed enzymatically after RNA transcription also according to CELLSCRIPT protocol. Cleanup of RNA during production steps was done using the RNeasy Mini Kit (QIAGEN) according to the manufacturer’s protocol for RNA Cleanup. CCR5-Uco-hetTALEN mRNAs production by BioNTech IMFS was performed from linearized plasmids with integrated poly(A)-tail. A 5′ ARCA cap was added to in vitro transcribed RNA. mRNA was purified using silica beads.

### Cell culture

Cell-culture material was purchased from Corning (Corning, NY), Greiner Bio One (Frickenhausen, Germany) and Sarstedt (Nümbrecht, Germany). HEK293T (ATCC CRL-3216), Jurkat (DSMZ, Braunschweig, Germany, ACC 282) and their derivatives were cultured in Dulbecco’s modified Eagle’s medium high-glucose Glutamax (Gibco, ThermoFisher) and RPMI Medium 1640 (Gibco, ThermoFisher), respectively. Both media were supplemented with 10% FCS, L-glutamine (2 mM), 100 U/ml penicillin and 100 μg/ml streptomycin. Cells were kept under standard conditions (37 °C, 100% relative humidity, 5% CO_2_).

Peripheral blood mononuclear cells (PBMCs) were isolated from fresh buffy coats, which are leftovers from erythrocyte concentrate production from whole-blood donations, using Ficoll (Biocoll, Merck, Darmstadt, Germany) density gradient centrifugation. All buffy coats used in these experiments were kindly provided by the Institute of Transfusion Medicine at the UKE after informed consent from healthy blood donors. If applicable, CD4^+^ cells were isolated from PBMC’s using human CD4 MicroBeads (Miltenyi Biotec, Bergisch Gladbach, Germany). Human T cells used in amplicon NGS experiments of homodimeric CCR5-Uco-TALEN were activated for 72 h with Dynabeads following the manufacturer’s instructions and cultured in X-VIVO 10 (Lonza, Basel, Switzerland) supplemented with 8% autologous plasma and 200 U/ml hIL-2. For all other experiments, human T cells were activated with CD3 and CD28 agonist conjugated beads, T-cell TransAct (Miltenyi Biotec), following the manufacturer. Human T cells were cultured in TexMACS Medium (Miltenyi Biotec) supplemented with 1-mM sodium pyruvate (Gibco, ThermoFisher), 50 µM 2-mercaptoethanol and 8% autologous plasma (if available) or 3% purchased human serum (Sigma-Aldrich, Steinheim, Germany). In addition, 200 U/ml hIL-2 or 155 U/ml recombinant human IL-7 and 290 U/ml recombinant human IL-15 (both Miltenyi Biotec) were added freshly to the medium. Cells were kept at 37 °C at 5% CO_2_.

### Electroporation

For electroporation, cells were washed in Opti-MEM I Reduced Serum Media (ThermoFisher) and resuspended to a concentration of 1 × 10^6^ primary human T cells/300 µl and 1.5 × 10^6^ Jurkat cells/600 µl in Opti-MEM. For single electroporation, 1–2 × 10^6^ cells were mixed with different amounts of CCR5-Uco-hetTALEN mRNA per arm in a 4-mm cuvette (BTX, Holliston, MA). Electroporation was performed with the Gene Pulser Xcell Electroporation System (Bio-Rad, Hercules, CA) using the following parameters: primary human T cells = 300 V, 1 square-wave pulse for 10 ms and Jurkat cells = 300 V, 1 square-wave pulse for 20 ms. Cells were kept at 32 °C and 5% CO_2_ for 24 h post electroporation [27].

### DNA and RNA isolation

Genomic DNA (gDNA) from sampled cells was isolated using the QIAamp DNA Blood Mini Kit (QIAGEN). Concentration of isolated DNA was assessed using the Qubit 2.0 Fluorometer together with the Qubit dsDNA BR Assay Kit (ThermoFisher). Total RNA was extracted from sampled cells using the RNeasy Mini Kit (QIAGEN) following the manufacturer’s spin protocol for animal cells. Homogenisation of cells was performed using QIAshredder spin columns (QIAGEN). The iScript Advanced cDNA Synthesis Kit for RT-qPCR (Bio-Rad) was used for reverse transcription. For each cDNA synthesis, 15 µl of total RNA (≤7.5 µg) were used as template. RNA concentration was determined with a Qubit 2.0 Fluorometer using the Qubit RNA HS Assay Kit (ThermoFisher).

### Real-time qPCR

All primers used in real-time qPCRs (RT-PCRs) and melting-curve analyses are listed in Table S9. Diluted CCR5-Uco-hetTALEN L + R plasmids were used to create standard curves for the calculation of copy numbers (Fig. S1). All RT-PCRs were done in triplicates.

Detection of CCR5-Uco-hetTALEN copy numbers in RNA isolates was performed with 2 µl template using the TB Green *Premix Ex Taq* (Tli RNaseH Plus) Kit from Takara Bio (Mountain View, CA) following the manufacturer’s protocol for the LC480 System. The following programme was used for amplification and determination of melting temperatures: 95 °C for 30 s, 40x [95 °C 5 s with ramp rate 4.4 °C/s, 60 °C 30 s with ramp rate 2.2 °C/s, single acquisition], 95 °C for 5 s, ramp rate 4.4 °C/s, 60 °C 60 s with ramp rate 2.2 °C, 95 °C ramp rate 0.11 °C/s with continuous acquisition, 50 °C 30 s, ramp rate 2.2 °C. CCR5-Uco-hetTALEN was detected after reverse transcription using primers hetTALENfw and hetTALENrv (detects mRNA and plasmid) or primers Kanfw and Kanrv (detects only plasmid) in a final concentration of 0.4 µM. Importin 8 (IPO8) was used as an external reference gene with the IPO8fw and IPO8rv primer pair in a final concentration of 0.4 µM. Crossing point (*C*_*P*_) calculations by LC480 software (version 1.5) for absolute quantification analysis was performed using the second derivative maximum method or using the fit points method if plasmid was detected. Plasmid copy numbers were calculated, if at least two of the three replicates showed the correct melting temperatures >89.0 °C.

Detection of CCR5-Uco-hetTALEN plasmid in gDNA isolates was performed with at least 20 ng template using the Maxima SYBR Green/Rox qPCR Master Mix (ThermoFisher) according to manufacturer’s (two-step) protocol.

### Droplet digital PCR (dPCR)

Droplet dPCR was performed using the Bio-Rad QX100 system. QuantaSoft 1.7.4.0917 was used to analyse data from dPCR. A detailed protocol for gene-editing frequency dPCR (GEF-dPCR) performance and analysis is available [28]. Primers and probes used for dPCR assays are provided in Table S9. The GEF-dPCR assay for *CCR5* gene-editing rates was performed with CCR5fw, CCR5rv, CCR5ref and CCR5mut primers and probes. For CCR2-specific GEF-dPCR primers and probes CCR2fw, CCR2rv, CCR5ref and CCR2mut were used. Double knockout of *CCR5* and *CCR2* (dKO) was assessed with CCR2fw, CCR5rv, CCR5ref, hEPORfw, hEPORrv and hEPORref primers and probes. Human erythropoietin receptor (hEPOR) was utilised as reference gene in all samples. To estimate copy-number variations (CNV) of *CCR5*, copy numbers of *CCR5* were compared to those of *hEPOR* with the following primers and probes: CCR5fw, CCR5rv, CCR5ref, hEPORfw, hEPORrv and hEPORref. CNV was calculated with QuantaSoft 1.7.4.0917. Large deletion at on-target *CCR5* and off-target *CCR2* were assessed with dPCR using hEPORfw, hEPORrv and hEPORref primers and probes, as well as the following primers and probes for the individual assay: dKO = CCR2fw, CCR5rv, CCR5ref; inversion = Inv1fw, CCR2rv, CCR5ref; integration = Int1fw, CCR2rv. 50–60 ng of DNA were employed for dPCRs.

### Lentiviral vector production and cell transduction

Lentiviral particles of third-generation vectors LeGO-CCR5-iB2-Puro+ and LeGO-CCR5^Δ55-60^-iB2-Puro+ were produced in accordance with standard protocols [26]. In short, 5 × 10^6^ 293T (ATCC CRL-3216, ATCC/LGC Standards, Wesel, Germany) cells were seeded and transfected with appropriate plasmids after overnight culture. Medium was exchanged after 6 h, and vector-containing supernatant was harvested and filtered 24 h later.

The production of Gibbon-ape-leukemia-virus-envelope (GALVenv) pseudotyped LeGO-S vectors encoding T-Sapphire used in the infection assay followed Mock et al. [29]. Viral supernatants with CCR5-tropic HIVenv (BaL-env) lentiviral particles encoding mCherry (LeGO-C) [26] were produced with 6 µg pcDNA3 BaL. Both viral supernatants were titrated on PM1 cells [30] in the presence of 8 µg/ml DEAE-dextran [26].

### Infection assay

The infection assay was performed 7 days post electroporation. For each transduction, 1 × 10^5^ electroporated cells (with or without CCR5-Uco-hetTALEN mRNA) were seeded in triplicates in 250 µl supplemented TexMACS Medium (3% human serum, 155 U/ml IL-7 and 290 U/ml IL-15) with 8 µg/ml DEAE-dextran into a 48-well culture plate. After addition of viral vector supernatants LeGO-S_GALVenv and LeGO-C_HIVenv to each well, cells were centrifuged at 1000 × *g* for 1 h at room temperature. Transduction rates were measured 4 days post-transduction at the BD LSRFortessa (BD Biosciences) using the following lasers and filters: mCherry 561 nm, filter 600 and 610/20; T-Sapphire 405 nm, filter 475 and 525/50. Mean LeGO-C_HIVenv transduction rates were normalized to GALVenv transduction rates.

### Proliferation assay

Proliferation of *CCR5*-edited and non-edited cells was monitored using the CellTrace CFSE Cell Proliferation Kit from Invitrogen. Three days after electroporation (with or without CCR5-Uco-hetTALEN mRNA) cells were stained with CellTrace CFSE dye in a concentration of 0.5 µM according to the manufacturer. Proliferation of stained cells was followed for 7 days. The cells were measured at the FACSCanto II (BD Biosciences). The following laser and filter combination was used for the measurements: CSFE = 488-nm laser, filters 502 and 530/30.

### Cytokine detection assay

Cytokine secretion (GM-CSF, IFN-α, IFN-γ, IL-2, IL-4, IL-5, IL-6, IL-9, IL-10, IL-12p70, IL-17A and TNF-α) was analysed using the MACSPlex Cytokine 12 Kit from Miltenyi Biotec. Media of TALEN-treated and non-treated control cells from different donors were harvested on days 6 and 12 post-activation and pooled from triplicates (electroporation of cells in biological replicates from each donor). Each pooled sample was measured in duplicates. Fifty microlitres of undiluted medium were used in each well. Cytokine concentrations were measured at the MACS-Quant Analyzer 10 Flow Cytometer (Miltenyi Biotec) using the Express Modes MACSPlex_Standard and MACSPlex_Sample. Flow-cytometry results were analysed using the MACSQuantify software version 2.13.1.

### Imaging flow cytometry (FC)

96 h after Jurkat cell transduction with LeGO-CCR5-iB2-Puro+ or LeGO-(CCR5^Δ55-60^)-iB2-Puro+, cells were harvested, washed with PBS, and resuspended in 250 µl PBS. Cells were fixed and permeabilised with the Inside Stain Kit (Miltenyi Biotec). Staining of cells was performed using 5 µl of the PerCP/Cy5.5 anti-human CD195 (BioLegend, San Diego, CA) and 5 µl of APC anti-human CD3 (Miltenyi) antibodies. After 15 min incubation in the dark at room temperature, cells were washed with PBS and resuspended in 100 µl fresh PBS. Cell images were obtained using the ImageStreamX Mk II System (Amnis/Luminex, Austin, TX); data were acquired and analysed with IDEAS Software package (Amnis/Luminex) using channels 5, 7, 11 and brightfield. Compensation was performed according to the software introduction using single-stained cells. BFP- (CCR5, or CCR5^Δ55-60^) positive cells were gated and their images investigated. Normal erode masque was applied to all images, and the internalization wizard was used to check the relative BFP to Cy5.5 signal localisation (Internalisation of BFP signal by Cy5.5 signal marking CCR5). Cells with internalised BFP signals were selected by choosing the cell population with an internalisation score ≥1.

### Flow cytometry (FC)

96 h after Jurkat cell transduction with LeGO-CCR5-iB2-Puro+ or LeGO-(CCR5^Δ55-60^)-iB2-Puro+, cells were harvested, washed with PBS, centrifuged and suspended in 100 µl PBS. Five microlitres of the PerCP/Cy5.5 anti-human CD195 (BioLegend) antibody were added. After 15-min incubation in the dark at room temperature, cells were washed with PBS and resuspended in 200 µl fresh PBS. Cells were measured for BFP and PerCP/Cy5.5 fluorescence at the BD FACSCanto II.

## Results

### Rates of mono- and biallelic knockout

**Fig. 1:**
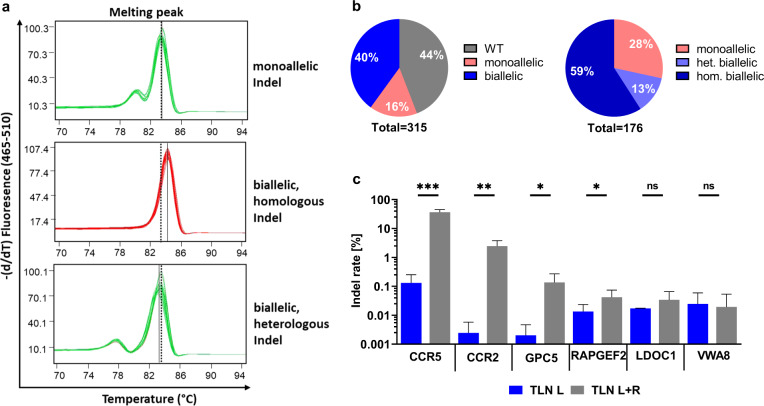
Results of single-cell high-resolution melting-curve analyses (scHRMCA) for the on-target *CCR5* locus and amplicon next-generation sequencing data for *CCR5* and potential off-targets. **a** Example of the melting-curve profiles and temperature (continuous line) of different Indel types (homologous editing in red and heterologous editing in green) compared to melting temperature of non-treated control cells (indicated by dotted line). –(d/dT) Fluorescence plotted against temperature in °C for monoallelic (15-bp deletion in one CCR5 allele), biallelic homologous (25-bp deletion in both CCR5 alleles) and biallelic heterologous (one short <5 bp and one larger 21-bp deletion) Indel. All melting profiles show results from seven cells measured in triplicates of Jurkat clones with the indicated Indels. **b** Relative distribution of CCR5-Uco-TALEN L + R edited single cells with wildtype (WT, grey), monoallelic (pink) and biallelic (blue) melting profiles (*n* = 315) or monoallelic (pink), heterologous (het., light blue) biallelic and homologous (hom., dark blue) biallelic editing as established by shHRMCA. 1 × 10^6^ primary T cells were electroporated with 5 µg of CCR5-Uco-TALEN L + R mRNA per arm. **c** Amplicon next-generation sequencing data from samples (*n* = 8) treated with homodimeric CCR5-Uco-TALEN (TLN L + R) and control samples treated with left CCR5-Uco-TALEN only (*n* = 2) for on-target *CCR5* and potential off-targets. Reads containing Indels at the TALEN binding sites were counted as Indel reads, while all other reads were considered non-edited. Indel rates were calculated using the ratio of reads containing Indels to all reads. Indel rates for *CCR5*, *CCR2*, *GPC5*, *RAPGEF2* and *LDOC1*. Black lines indicate the mean. Statistical analysis of Indel ratio was done using a one-tailed Welch’s *t*-test with a confidence interval of 95%. *P* values: ns *p* > 0.1234, *p < 0.0332, ***p < 0.0001.

Protecting CD4^+^ T cells against infection with CCR5-tropic HIV strains is possible via knockout of the HIV co-receptor CCR5. However, to obtain CCR5-negative cells, efficient knockout of both alleles will be required. To assess, whether CCR5-Uco-TALEN activity leads to gene editing at both alleles, we developed (Figs. 1a and S2) and applied a new protocol for scHRMCA. The method is based on previous observations that indels in a PCR amplicon lead to detectable shifts in its melting temperature. In addition, the presence of two different fragments results in formation of heteroduplexes, which changes appearance of the melting curve [31–33]. Quality of genome editing caused by CCR5-Uco-TALEN cleavage was analysed in total for 315 single cells from two independent electroporations (5 µg CCR5-Uco-TALEN L + R mRNA per arm and 1 × 10^6^ cells). Melting temperatures and curves of treated cells were compared to those of non-edited cells (Fig. 1a). In total, 139 of the analysed cells (44%) showed melting-temperature profiles of non-edited, i.e., WT cells, 50 cells (16%) had monoallelic (indel found in one allele) melting-curve profiles, and 126 (40%) biallelic (indels found in both alleles) ones (Fig. 1b). Melting-curve profiles of biallelic gene editing were further divided into homologous (two alleles harbouring identical indels) and heterologous (two alleles harbouring different indels) biallelic editing. Altogether, 71.6% of edited cells showed gene editing at both *CCR5* alleles. Surprisingly, most of those cells showed homologous melting profiles (Fig. 1b).

### CCR5-Uco-TALEN off-target activity

Low *CCR2* off-target activity as compared to other *CCR5*-directed designer nucleases was established for CCR5-Uco-TALEN, previously [18]. To obtain a more comprehensive picture, we here determined potential off-targets of the CCR5-Uco-TALEN by in silico analysis and performed amplicon NGS for ten of the identified loci (*CCR2*, *MUC16*, *VWA8*, *KIRELL*, *GPC5*, *IQSEC2*, *BRS3*, *LDOC1*, *RAPGEF2*, *SEMA3C*), as well as for the on-target *CCR5* (Table S5). Depth of sequencing ranged from >4.000 to >65.000 reads (Fig. S3) per target and sample. As expected, high editing levels were found at the on-target (*CCR5*) site for samples treated with both CCR5-Uco-TALEN arms (Fig. 1c). In contrast, we saw no evidence for TALEN activity for five out of the ten potential off-targets (*SEMA3C*, *KIRELL*, *MUC16*, *BRS3*, *IQSEC2*) (Fig. S4). In accord with previous data [18], definite Indel rates (1.1–4.8%) were observed at the established off-target *CCR2* for samples treated with both CCR5-Uco-TALEN arms (Fig. 1c). At loci *GPC5* and *RAPGEF2*, low, but significant levels of gene editing at the TALEN binding site were detected in some, but not all CCR5-Uco-TALEN L + R-treated samples. Deletions >4 bp in single samples for *LDOC1* and *VWA8* were observed for CCR5-Uco-TALEN L + R-treated samples, as seen for on-target *CCR5* and off-targets *GPC5*, *CCR2* and *RAPGEF2* (Fig. S5). However, Indel ratios for *LDOC1* and *VWA8* were non-significant (Fig. 1c).

### Optimisation of CCR5-Uco-TALEN for clinical use

**Fig. 2:**
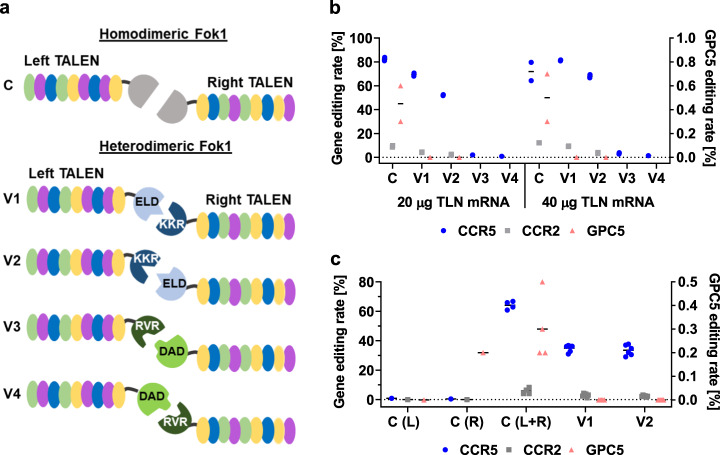
The activity of different CCR5-Uco-hetTALEN variants. **a** Comparison of different TALEN variants containing codon-optimised homo- and obligatory heterodimeric Fok1 nucleases. C = homodimeric variant. V1 = ELD left TALEN, KKR right TALEN. V2 = KKR left TALEN, ELD right TALEN. V3 = RVR left TALEN, DAD right TALEN. V4 = DAD left TALEN, RVR right TALEN. **b** Gene-editing rates in Jurkat cells for different CCR5-Uco-hetTALEN variants. Jurkat cells were electroporated with 20 µg (10 µg per arm) and 40 µg (20 µg per arm) of self-made CCR5-Uco-TALEN mRNA per 1.5 × 10^6^ for each of the indicated Fok1 variants in duplicates. Editing rates were assessed by GEF-dPCR for on-target *CCR5* (blue circle), off-target *CCR2* (grey square) and *GPC5* (pink triangle) in Jurkat cells (*n* = 2). Note different scale for GPC5. **c** Combined data for on- and off-target editing at *CCR5* (blue circle), *CCR2* (grey square) and *GPC5* (pink triangle) loci in primary T cells for the homodimeric (*n* = 4) and the two obligatory heterodimeric variants V1 (*n* = 5) and V2 (*n* = 6). 1 × 10^6^ primary human T cells were electroporated with 5 µg of CCR5-Uco-hetTALEN mRNA per arm. Note different scale for GPC5.

**Fig. 3:**
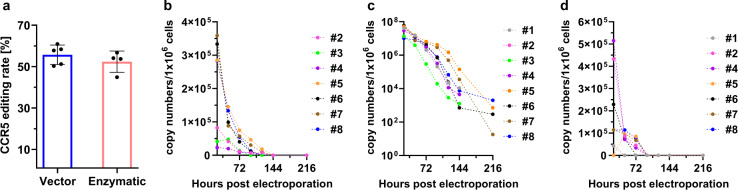
Influence of different polyadenylation strategies and CCR5-Uco-hetTALEN decay in primary human T cells. **a** Comparison of mRNA transcribed from a plasmid containing poly(A) sequence and mRNA with enzymatically added poly(A). *CCR5* gene-editing rates of 1 × 10^6^ primary human T-cell electroporated with 5 µg of CCR5-Uco-hetTALEN mRNA per arm with different poly(A) adding. Error bars show SD values. **b**–**d** CCR5-Uco-hetTALEN copy numbers monitored by qPCR up to 144–216 h post electroporation. Primary T cells from different donors were treated with 3 µg of CCR5-Uco-hetTALEN_poly(A) mRNA per 2 × 10^6^ cells. qPCR was performed in triplicates for each sample and diluted standard. Copy-number calculation was based on *C*_*p*_ values from a standard curve with defined TALEN plasmid copies. **b** Detection of CCR5-Uco-hetTALEN copy numbers after cDNA synthesis of total RNA extracted from eight different treated samples. **c** Detection of residual CCR5-Uco-hetTALEN plasmid used in mRNA production in RNA samples of seven CCR5-Uco-hetTALEN mRNA-treated donors. **d** Detection of residual CCR5-Uco-hetTALEN plasmid used in mRNA production in gDNA samples of seven CCR5-Uco-hetTALEN mRNA-treated donors.

TALEN (or any other nuclease) activity at off-target sites is correlated with a higher risk of unwanted genomic changes. Therefore, to translate genome editing towards clinical application, potential risks associated with off-target activities need to be minimised. As evident from Table S5, all but one (*CCR2*) TOP10 in silico predicted off-targets of CCR5-Uco-TALEN were due to binding and cleavage of two identical TALEN arms (in eight cases two right, in one case two left arms) and homodimerisation of Fok1. To exclude this phenomenon and thus drastically reduce potential off-target sites, we aimed to replace the homodimeric Fok1-cleavage domain of the CCR5-Uco-TALEN with an obligatory heterodimeric Fok1 variant. To this end, we generated novel, codon-optimised Fok1 variants introducing previously described mutations, namely ELD/KKR and RVD/DAD [25]. We tested both obligatory heterodimeric variants fused to left and right TALEN arms and vice versa. To address any potential impact of codon optimisation, we also generated a codon-optimised homodimeric (WT) Fok1-cleavage domain (Fig. 2a). 1.5 × 10^6^ Jurkat T cells were electroporated in duplicates with (2 × 10 µg) 20 μg and (2 × 20 µg) 40 μg of mRNA encoding one of the five different TALEN variants (four obligatory heterodimeric, one homodimeric) and analysed for gene-editing events at the *CCR5* locus using GEF-dPCR (Fig. 2b). *CCR5* gene-editing rates of 82.4 ± 1.4% for 20 µg of mRNA and 71.9 ± 7.6% for 40 µg of mRNA were measured for WT, homodimeric Fok1 TALEN. Both heterodimeric variants 1 (ELD left/KKR right) and 2 (KKR left/ELD right) showed on-target gene-editing rates above 50% with variant 1 facilitating much higher activity, whereas variants 3 (RVR left/DAD right) and 4 (DAD left/RVR right) mediated only low gene-editing frequencies of up to 3.4%. Variants 1 and 2 were further analysed for gene-editing frequencies at off-targets *CCR2* and *GPC5* by GEF-dPCR (Fig. 2b). As expected, *CCR2* off-target editing (caused by binding of left and right TALEN arms) was still present for the obligatory heterodimeric Fok1 variants. In striking contrast, however, both heterodimeric Fok1 variants mediated no editing at the homodimeric off-target *GPC5* anymore indicating that homodimerisation had successfully been prevented. Subsequently, we tested the two obligatory heterodimeric TALEN variants 1 and 2 as compared to the homodimeric CCR5-Uco-TALEN in primary human T lymphocytes. To do so, we electroporated 1 × 10^6^ activated primary T cells with (2 × 5 µg) 10 µg mRNA encoding the different TALEN variants. As depicted in Fig. 3d, inclusion of obligatory heterodimeric Fok1 variants resulted in up to twofold lower on-target activity as compared to homodimeric CCR5-Uco-TALEN (Fig. 2c). More importantly, we again observed no off-target activity at the *GPC5* locus for the heterodimeric variants. Heterodimeric TALEN variant 1 was chosen for future characterisation and application; hereafter, it will be referred to as CCR5-Uco-hetTALEN.

### Optimisation of mRNA in vitro production for clinical use

Beside TALEN specificity and activity, its way of delivery to target cells represents a very important aspect of TALEN-based treatment. The mRNA electroporation is an easy and feasible method commonly used on laboratory scale. Up-scaling of mRNA electroporation for clinical use requires not only large amounts of mRNA, but also its cost-effective and reliable production. Whereas the polyadenylation step during mRNA production can readily be performed enzymatically, the length of the generated poly(A) tail cannot be controlled and might differ from batch to batch. Furthermore, enzymatic polyadenylation substantially increases production costs. Therefore, we tested performance of a 120-nt long poly(A) sequence already integrated into the CCR5-Uco-hetTALEN plasmids behind the open reading frame to allow direct production of polyadenylated RNA from plasmid. 1 × 10^6^ activated primary human T cells were electroporated with (2 × 5 µg) 10 µg CCR5-Uco-hetTALEN L + R mRNA with enzymatic polyadenylation (enzymatic) or poly(A) transcribed from production plasmid (vector). *CCR5*-editing rates were determined by GEF-dPCR. Both CCR5-Uco-hetTALEN mRNA productions showed efficient *CCR5* editing of 55.7 ± 4.7% for plasmid-derived poly(A) and 52.4 ± 5.1% for enzymatic polyadenylation, respectively (Fig. 3a).

### CCR5-Uco-hetTALEN mRNA kinetics

Continuous presence of exogenous proteins after infusion of ex vivo modified T cells might induce host immune reactions. Consequently, fast clearance of CCR5-Uco-hetTALEN mRNA is important. We monitored copy numbers of CCR5-Uco-hetTALEN in RNA isolates using real-time quantitative PCR for TALEN-treated samples. To this aim, activated human CD4^+^ T cells from seven different donors were treated with 3 µg of CCR5-Uco-hetTALEN_poly(A) L + R mRNA per 2 × 10^6^ cells. 0.5 × 10^6^ cells were harvested before and every 24 h post electroporation. About half of the extracted total RNA was used for cDNA synthesis, and 10% of cDNA were then used as template for RT-qPCR to detect CCR5-Uco-hetTALEN. Importin 8 was amplified as reference gene. Based on the cDNA amount used as a template for RT-qPCR, calculated copy numbers correspond to app. 25.000 cells (assuming loss-free RNA isolation and cDNA synthesis). *C*_*P*_ values for IPO8 revealed only minor differences between donor samples and time points, whereas TALEN *C*_*P*_ values showed larger variations (Fig. S6a). Early after electroporation (24 h), TALEN copy numbers showed high inter-sample variance (1 × 10^7^–6 × 10^7^ copies per 1 Mio cells) (Figs. 3b and S6b). Thereafter, copy numbers decreased rapidly and were low at 216 h post-electroporation with the highest calculated copy number being 2000 CCR5-Uco-hetTALEN mRNA copies per 1 Mio treated cells.

### CCR5-Uco-hetTALEN encoding plasmid

Large amounts of mRNA can be easily produced by in vitro transcription from a plasmid template, as also described above for CCR5-Uco-hetTALEN. After the transcription step, during mRNA production, the donor plasmid is removed. Nevertheless, small amounts of plasmid could remain in the mRNA preparation and might have a negative impact on transfected cells. Hence, we developed a real-time PCR to specifically detect residual donor plasmid in RNA and DNA isolated from CCR5-Uco-TALEN L + R mRNA-treated samples. To do so, we used gDNA (20 ng) and RNA from samples of seven different donors treated with 3 μg of CCR5-Uco-hetTALEN_poly(A) L + R mRNA per 2 × 10^6^ CD4^+^ T cells, as in previous experiments. Plasmid copy numbers dropped rapidly after electroporation, and in all tested samples, no CCR5-Uco-hetTALEN plasmids were detected 144 h post-electroporation (Fig. 3c, d). Reduced plasmid copy numbers in gDNA isolates are probably due to lower template concentration. Amounts of gDNA used in RT-PCRs equal app. 2900 cells.

### Off-target activity of CCR5-Uco-hetTALEN

**Fig. 4:**
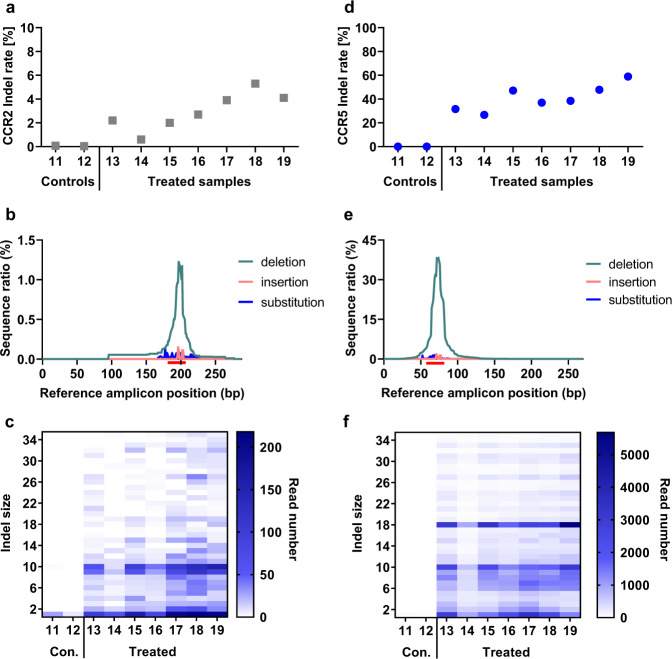
Amplicon next-generation sequencing data for off-target *CCR2* and on-target *CCR5*. **a** *CCR2* Indel rates as established by amplicon NGS for small-scale samples. Data are shown for two control samples and seven CCR5-Uco-hetTALEN-treated samples. **b** Example (sample 18) of the distribution of mutation position calculated with CRISPRESSO2 for samples treated with CCR5-Uco-hetTALEN L + R. Numbers of reads with deletions, insertions and substitutions found for *CCR2* based on position in reference amplicon plotted with GraphPad Prism 8.4.3. **c** The number of *CCR2* Indel containing reads as found by amplicon NGS plotted against gap size. **d** *CCR5* Indel rates obtained from amplicon NGS data analysis. The graph shows Indel rates found in DNA isolated from two control samples and seven CCR5-Uco-hetTALEN-treated samples. **e** Example (sample 18) of the distribution of mutation position calculated with CRISPRESSO2. The number of reads with deletions, insertions and substitutions found after alignment with *CCR5* based on position in reference amplicon plotted with GraphPad. **f** Numbers of *CCR5* Indel reads from amplicon NGS data plotted based on Indel size.

As described above, translation of gene-editing tools towards clinical application requires risk assessment associated with off-target activities. However, off-targets analysed above (except *CCR2*) were caused by homodimeric TALEN binding. Consequently, to empirically study the potential off-target activity of optimised CCR5-Uco-hetTALEN, we performed a novel in silico analysis exclusively focussing on heterodimers. For subsequent NGS analysis, we preferentially selected intragenic off-targets (Table S6). Potential targets of CCR5-Uco-hetTALEN chosen for NGS analysis were: *CCR5*, *CCR2*, *CXCR6*, *GLP1R*, *CACNA1B*, *ASIC*, *SAMD12*, *ADYC2*, *PGC*, *MAT2B* and *UBXN10*. Again, we used amplicon NGS and obtained 10,000 to >100,000 reads for most targets and samples, except for potential off-targets *PGC* and *UBXN10*, which were excluded from statistical analysis (Fig. S7). In accord with GEF-dPCR data, Indels were found in off-target *CCR2* in CCR5-Uco-hetTALEN_polyA L + R mRNA-treated samples (Fig. 4a). As expected, most Indels were deletions, a small fraction of reads showed insertions, and only very few substitutions were detected, as exemplarily shown in Fig. 4b for one of the CCR5-Uco-TALEN L + R-treated samples. Indels found with the highest frequencies within *CCR2* were 1-bp deletions, and deletions of 10 or 9 base pairs. The vast majority of deletions in the *CCR2* gene were smaller than 15 base pairs (Fig. 4c). Total Indel read counts for all other investigated off-targets were low, and no significant differences to non-treated control samples were seen for *ADCY2*, *CACNA1B*, *CXCR6*, *MAT2B*, *ASIC*, *GLP1R* and *SAMD12* (Fig. S8).

### On-target activity of CCR5-Uco-hetTALEN

**Fig. 5:**
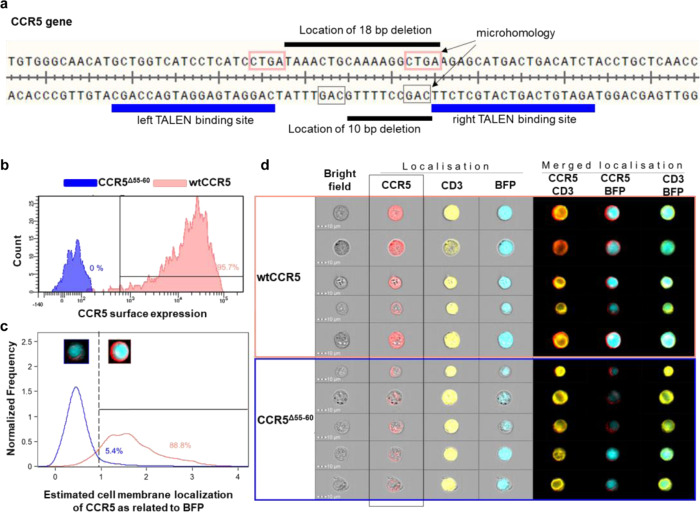
The most abundant 18-bp deletion in *CCR5* is MMEJ-induced and results in a protein variant (CCR5^Δ55-60^) with impaired cell-surface expression. **a** Microhomologies found at the positions of the most frequent 18- and 10-bp deletions at the *CCR5* locus indicating microhomology-directed repair. **b**–**d** CCR5-negative cells transduced with lentiviral vectors encoding BFP and either wildtype (wt) CCR5 or CCR5^Δ55-60^ were analysed for CCR5, CD3 and BFP expression. **b** CCR5 cell-surface expression. >95% of BFP-positive cells expressing wtCCR5, but no BFP-positive cells containing the CCR5^Δ55-60^ variant became positive after cell-surface staining with anti-human CCR5-PerCP-Cy5.5 antibody. **c** Fixed and permeabilised Jurkat cells expressing either wtCCR5 or CCR5^Δ55-60^ were stained with anti-human CCR5-PerCP-Cy5.5 and anti-human CD3-APC antibodies. Over 1000 images obtained with imaging flow cytometry were used to estimate cell membrane localisation of both CCR5 variants. CCR5 signal localisation was related to BFP present in the cytoplasm. With the gate set at 1, cell membrane localisation of wtCCR5 and CCR5^Δ55-60^ was found for 88.8% and 5.4%, respectively. **d** Examples of Image Stream images of the fixed and permeabilised Jurkat cells expressing either wtCCR5 or CCR5^Δ55-60^ stained with anti-human CCR5-PerCP-Cy5.5 and anti-human CD3-APC. CCR5, as a membrane protein, should be localised mainly outside of the BFP with cytoplasmic localisation.

Upon binding CCR5-Uco-hetTALEN induces double-strand breaks (DSBs) at the target site. Those DSBs are repaired by different cellular mechanisms, particularly non-homologous end-joining (NHEJ)  and microhomology-mediated end-joining (MMEJ), often leading to Indels of different sizes. To characterise CCR5-Uco-hetTALEN-generated Indels, we also performed amplicon NGS at the on-target *CCR5*. As expected from GEF-dPCR data, Indels were found at on-target *CCR5* at high frequencies (26.7–58.9%) in CCR5-Uco-hetTALEN-treated samples (Fig. 4d and Table S7). The majority of Indel reads at the *CCR5* locus contained deletions, which peaked around the probable cut-site between the two TALEN binding sites (Fig. 4e). Analysis of Indel sizes showed that most edited clones belonged to one of three groups with 18- (14.6%), 10- (9.3%) and 1-bp (10.0%) Indels (Fig. 4f). Deletions of 18 and 10 base pairs were found between gene positions containing microhomologies (Fig. 5a). The 18-bp deletion does not induce a frameshift, but the loss of six amino acids localised in the intracellular loop 1 (ICL1) [18]. Therefore, we wondered whether this deletion does have any functional consequences. Modelling of the CCR5 protein containing the 18-bp deletion (CCR5^Δ55-60^) indicated correct folding of the protein into seven α-helices, but comparison to WT CCR5 revealed, as expected, a shorter ICL1 of CCR5^Δ55-60^ (Fig. S9). To test the functional impact of the deletion, we cloned LeGO vectors encoding either WT CCR5 (LeGO-CCR5-iB2-Puro+) or CCR5^Δ55-60^ (LeGO-CCR5^Δ55-60^-iB2-Puro+), each in conjunction with BFP and puromycin resistance, and transduced CCR5-negative T cells with either of the CCR5 variants. Expression and localisation of CCR5 variants at the cell surface of transduced cells (as identified based on BFP expression) were measured after antibody staining by flow cytometry (FC) and imaging flow cytometry (Image-FC). By classical FC analysis, essentially no CCR5 was found on the surface of live, BFP-positive cells transduced with LeGO-CCR5^Δ55-60^-iB2-Puro+ (Fig. 5b). In striking contrast, >95% of cells transduced with LeGO-CCR5-iB2-Puro+ displayed CCR5 on their surface. Importantly, analysis of multiple individual pictures obtained by Image-FC revealed that CCR5^Δ55-60^ was expressed in transduced cells, but largely retained in the cytoplasm. Quantitative evaluation indicated that cell-surface localisation of CCR5 was observed in app 5% of CCR5^Δ55-60^-expressing cells, only, in contrast to almost 90% for CCR5 WT (Fig. 5c, d).

### Gene-editing kinetics at on- and off-target loci

**Fig. 6:**
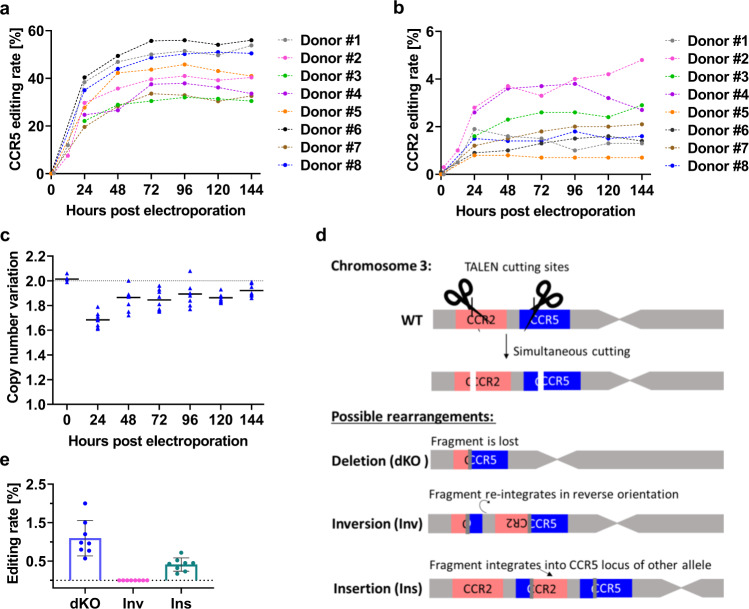
Monitoring of CCR5-Uco-hetTALEN activity at on-target CCR5 and off-target CCR2 in primary human T cells. Primary human CD4^+^ T cells from eight different donors were treated with 3 µg of CCR5-Uco-hetTALEN_poly(A) L + R mRNA per 2 × 10^6^ cells. **a** Gene-editing kinetics of CCR5 monitored over 6 days. Data were obtained using GEF-dPCR. **b** Gene-editing kinetics of *CCR2* monitored over 6 days. Data were obtained using GEF-dPCR. **c** Copy-number variation (CNV) between *CCR5* and *hEPOR* determined by dPCR. Black line shows mean. The dotted line at CNV of 2.0 indicates equal copy numbers between measured targets. **d** Schematic diagram of chromosome 3 with location of CCR5-Uco-hetTALEN binding sites at loci *CCR2* and *CCR5*. Most likely possible chromosomal rearrangements due to simultaneous cutting at both loci and excision of a 15-kb fragment: (dKO) removal of whole 15-kb fragment and re-ligation of the chromosome. (Inv) Inversion and re-integration of the 15-kb fragment. (Ins) Insertion of the 15-kb fragment into the *CCR5* locus at the other allele. Dark grey bars indicate possible Indels at repaired sites. **e** Deletion (dKO, marked with blue), inversion (inv, marked with pink) or integration (ins, marked with green) of the 15-kb fragment between *CCR5* and *CCR2* due to CCR5-Uco-TALEN activity. dPCR results for treated samples from seven different donors at 144 h post electroporation. Error bars show SD values.

Knowing the actual kinetics of the gene-editing process at both the on- and off-target sites is important for several reasons. Obviously, to allow correct characterisation of the final product, the editing process needs to be finished by the time of application to patients. Also, as soon as the editing is accomplished, in-process quality controls are possible. Finally, studying editing kinetics might provide important insights into the underlying biology and any impact of donor-specific characteristics thereon. To address these questions, we electroporated 2 × 10^6^ activated human CD4^+^ T cells of eight different donors with 3 µg CCR5-Uco-hetTALEN_poly(A) mRNA per arm and monitored gene-editing rates over a period of 6 days by GEF-dPCR. 144 hours post electroporation, treated cells showed a mean *CCR5* editing rate of 42 ± 10%. *CCR2* gene-editing rates were in the range of 2.2 ± 1.3% on average (Fig. 6a, b) revealing high donor variability. Interestingly, we observed different editing kinetics at the on-target and off-target loci. Indeed, in all individual samples *CCR5* editing occurred mainly up to 72 h post electroporation (most efficiently within the first 48 h). In contrast, *CCR2* editing was completed in 48 h, in several samples even at 24 h post electroporation.

An alternative way to document ongoing editing is the measurements of copy-number variants (CNVs) between *CCR5* and a diploid reference gene, in our experiment the *hEPOR*. Duplex *CCR5* dPCR data for the eight samples showed pronounced CNVs (1.6–1.7) in all TALEN-treated samples at 24 h post-electroporation. Copy numbers of the two genes converged at 48 h post-electroporation but remained decreased for *CCR5* as compared to *hEPOR* during the whole observation period (Fig. 6c). Importantly, the observed decrease in *CCR5* copy numbers could be due to two different phenomena. Early after transfection, it most probably reflects ongoing nuclease activity resulting in DSBs that prevent amplification. At later time points, the lower *CCR5* copy numbers indicate the presence of deletions involving target sites of the *CCR5* probe and/or primers used in dPCR.

### Detection of specific large deletions at the *CCR5* locus

The *CCR5* and *CCR2* genes are neighbouring genes on chromosome 3 with a distance of app. 10 kb between the protein coding regions. Resultantly, simultaneous induction of DSBs at both loci (on- and off-target) is associated with a definite likelihood of chromosomal rearrangements, most probably 15-kb deletions (fragment size between CCR5-Uco-hetTALEN *CCR5* and *CCR2* cut sites), but principally also inversions or integrations (Fig. 6d). To study this, we investigated three scenarios—deletion of the 15-kb fragment between the two TALEN binding sites at *CCR5* and *CCR2*, integration of the cut-out 15-kb fragment into the induced DSB in *CCR5* locus (of the other chromosome), and inversion of the 15-kb fragment. To estimate frequencies of these events, we performed dPCR on samples from gene-editing kinetics experiments at 144 h post-electroporation treated with CCR5-Uco-hetTALEN. Ratios of deletion (dKO), integration (int) or inversion (inv) were calculated according to copy numbers of the reference gene (*hEPOR*). We were able to detect deletion of the 15-kb fragment (dKO), which occurred at a mean frequency of 1.1 ± 0.5% in tested samples. No inversion events were detected. However, integration of the 15-kb fragment into the CCR5-Uco-hetTALEN cut-site at the *CCR5* locus was found at a mean ratio of 0.4 ± 0.2% (Fig. 6e).

### Mono- and biallelic editing in CCR5-Uco-hetTALEN-treated cells

**Fig. 7:**
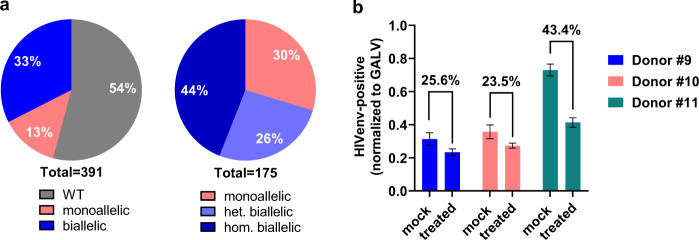
scHRMCA data and HIV susceptibility of *CCR5*-edited CD4^+^ T cells. Primary human CD4^+^ T cells from three different donors (#9–#11) were electroporated with 2.5 μg of CCR5-Uco-hetTALEN_polyA L + R mRNA per 1 × 10^6^ cells or without mRNA. **a** scHRMCA results showing wildtype (WT, grey), mono- (pink) and biallelic (blue) gene editing at the *CCR5* locus, as well as the percentage of homologous (hom., dark blue) and heterologous (het., light blue) biallelic editing. **b** HIV infection assay with pseudotyped lentiviral vectors LeGO-GALVenv and LeGO-HIVenv simultaneously. Measurement of fluorescent positive cells was performed by flow cytometry. Bar graph shows HIVenv positive cells normalized to GALVenv positive cells for donor #9 (blue), #10 (pink) and #11 (green) for *CCR5*-edited (treated) and non-edited (mock) cells.

In order to determine mono- and biallelic editing at the *CCR5* locus, scHRMCA was performed on *CCR5*-edited cells treated with CCR5-Uco-hetTALEN from donors #9–#11. In total, 54% of analysed cells showed a melting profile of WT cells, while 13% were monoallelic and 33% biallelic edited at the *CCR5* locus. As for the homodimeric CCR5-Uco-TALEN, we observed high rates of biallelic relative to monoallelic editing of 70% (relative to all edited cells). Again, the majority of biallelic editing had a homologous melting profile (Fig. 7a).

### CCR5 expression by gene-edited CD4^+^ T cells

To verify functional receptor knockout, CCR5 surface expression was measured 7 days post-electroporation and compared between *CCR5*-edited and non-edited cells. Cells from three different donors (#9–#11) were stained for viability (7AAD) and CCR5 surface expression. Comparison of viable CCR5^+^ cells in treated and non-treated cells showed a reduction of CCR5 expression of 22.0% for donor #9, 35.3% for donor #10 and 44.3% for donor #11 (Fig. S10a+b). Importantly, loss of CCR5 surface expression requires complete gene knockout. Thus, the observed reductions are in accord with the GEF-dPCR and scHRMCA results shown above.

### Functional analysis of *CCR5*-edited cells

In a further set of experiments, we aimed to check if *CCR5*-edited cells maintain their functional capacities. To this end, we compared proliferation, cytokine production and the susceptibility towards HIV infection of untreated vs. CCR5-Uco-hetTALEN-treated cells. Resistance against HIV was tested in a BSL-2 transduction assay with pseudotyped lentiviral vectors (HIVenv and GALVenv) encoding different fluorescent markers. *CCR5*-edited and non-edited cells from three different donors (#9–#11) were transduced simultaneously with GALVenv- and HIVenv-pseudotyped lentiviral vectors in triplicates. HIVenv-transduced were normalised to GALVenv-transduced cells. HIVenv transduction rates of CCR5-edited cells were reduced by 25.6% and 23.5% for donors #9 and #10, when compared to non-edited cells. In line with FC data, donor #11 showed the highest reduction of HIV susceptibility (43.4%) for *CCR5*-edited cells (Fig. 7b).

It could not be excluded that gene-editing events influenced the T-cell functionality. Therefore, we compared proliferation and cytokine secretion of *CCR5*-edited and non-edited control cells in two different assays. Proliferation of cells was monitored over 7 days for three different donors (#9–#11) after staining with the dye CellTrace CSFE by FC. Histogram overlays of the measured fluorescent intensities showed identical patterns for CCR5-edited and non-edited cells (Fig. S11). Hence, editing of the *CCR5* locus had no major impact on cell proliferation. Moreover, secretion of 12 different cytokines (GM-CSF, IFN-α, IFN-γ, IL-2, IL-4, IL-5, IL-6, IL-9, IL-10, IL-12p70, IL-17A and TNF-α) was determined in media of CCR5-edited and non-edited CD4^+^ T cells from three different donors (donors #9–#11). All tested conditions showed high secretion of GM-CSF and IFN-γ (Fig. S12), whereas concentrations of IL-2 and IFN-α were low. Except for IL-4, IL-10 and IL-12p70, all other cytokine concentrations were higher at day 6 after T-cell activation compared to day 12. No difference was observed in concentrations of secreted cytokines between *CCR5*-edited and non-edited cells.

## Discussion

Novel, ideally curative treatment options for HIV are still needed. CCR5 has emerged as a preferred target, particularly for gene-therapeutic strategies, not the least in view of successful HIV elimination in two patients after transplantation of CCR5-negative HSCs [14, 15]. A number of groups, including our own [18, 19], have developed designer nucleases that target *CCR5* to endow gene-modified cells with resistance to CCR5-tropic HIV [34–41].

We previously demonstrated that a CCR5-specific TALE nuclease developed in our group, CCR5-Uco-TALEN, mediates efficient *CCR5* knockout in primary human T cells [18]. Based on our observation that off-target activity of CCR5-Uco-TALEN was mainly due to homodimers of identical TALEN arms, we have now optimised this TALEN by introducing an obligatory heterodimerising Fok1 domain. Using this safety-optimised CCR5-Uco-hetTALEN and mRNA transfection, we obtained *CCR5* editing rates of app. 50% in primary T cells in the small-scale experiments reported here. Higher editing efficiencies were possible but associated with substantially increased off-target activity at the highly similar *CCR2* locus. Our editing rates are well in line with previous [18] and novel data [41] for selected, high-efficiency TALENs. In comparison to zinc finger nucleases, which were already tested in a clinical study, our CCR5-Uco-hetTALEN showed higher *CCR5*-editing rates with better safety profile [40, 42]. Efficiency and specificity of all CCR5-specific designer nucleases depend on the nuclease class, target cell type and method of system delivery. *CCR5* disruption reaches up to 60–80% in primary CD4^+^ T cells edited ex vivo after mRNA electroporation. Notably, in comparison to other CCR5-targeting designer nucleases, the *CCR2* off-target activity of CCR5-Uco-TALEN was previously shown to be low [18].

To ensure protection of T cells from HIV infection, complete *CCR5* knockout is essential. We here for the first time report, a new method to determine biallelic Indels in individual cells based on single-cell HRMCA. We observed that with both homodimeric and heterodimeric CCR5-Uco-TALEN, more than 70% of edited cells harboured Indels in both alleles. This data is in accord with reports from other groups [35, 37, 40] but higher than for some programmable nucleases reported [34, 43, 44]. Interestingly, most biallelic edits were homologous indicating homology-directed repair using the sister chromosome [45] as a main mechanism. We are not able to exclude wrong calling of some of the tested cells, e.g. due to large deletions found in one allele, but assume this number to be small. Indeed, NGS data at the *CCR5* locus indicated that most Indels were <30 bp and thus detectable in our single-cell HRMCA.

Previously, off-target activity of TALENs has been shown to be relatively low as compared to other types of nucleases [18, 37]. Two of the nine in silico predicted loci for the original, homodimeric CCR5-Uco-TALEN, *CCR2* and *GPC5*, showed a significant off-target activity, while three further loci, *VWA8*, *RAPGEF2* and *LDOC1* revealed elevated Indel read counts with deletions in single samples. Remarkably, except for *CCR2*, all in silico predicted TOP10 off-targets were induced by binding of homodimers of two identical TALEN arms. To further reduce the risk of off-target cutting, we replaced the classical Fok1 nuclease in CCR5-Uco-TALEN with an obligate heterodimeric Fok1 developed by Doyon et al. [25]. At equal mRNA concentrations, we observed slightly reduced on-target activity of the new variant, CCR5-Uco-hetTALEN, as compared to CCR5-Uco-TALEN. However, after adjustment of the applied mRNA concentrations, comparable on-target activity could be ensured for heterodimeric CCR5-Uco-hetTALEN. More importantly, CCR5-Uco-hetTALEN indeed showed remarkably improved target specificity. In fact, using sensitive dPCR, we did not observe any Indel formation at the previously identified off-target *GPC5* anymore. Moreover, using amplicon NGS, we found no Indels at any of the newly in silico predicted off-targets for the CCR5-Uco-hetTALEN.

Simultaneous cutting at both the *CCR5* on-target and the *CCR2* off-target sites (located next to each other on chromosome 3) might induce chromosomal aberrations, as also reported by other groups with all types of programmable nucleases [46–48]. Therefore, we designed and applied specific dPCR assays to quantify the incidence of 15-kb deletions and possible translocations between the *CCR2* off-target and *CCR5* on-target binding sites of CCR5-Uco-hetTALEN. Complete deletion (dKO) and translocations of the 15-kb fragment (Ins) were observed at low frequencies, only. However, particularly dKO frequencies can be expected to increase with raising *CCR2* off-target activity, which highlights the importance of a short and well-dosed nuclease expression, as warranted by mRNA electroporation.

Unfortunately, in many studies, actual off-target activities of CCR5-directed designer nucleases were not well-characterised even at the *CCR2* locus thus precluding direct comparison. It is of note, however, that first clinical studies on ZNF-mediated *CCR5* disruption showed safety and feasibility [38] and did not provide any evidence of severe side effects, despite the fact that preclinical studies with the used ZFN reported off-target rates at *CCR2* gene of >20% [40]. Current clinical studies investigate safety of *CCR5*-edited CD34^+^ cells treated with zinc finger nucleases and CRISPR/Cas nucleases (ClinicalTrails.gov NCT03164135 [49] and NCT02500849 [40]).

The aberrations induced by parallel cutting at both *CCR5* and *CCR2*, mostly 15-kb deletions, as well as the simultaneous knockout of both genes could be expected to decrease the functionality of affected T cells, particularly if both alleles are destroyed [18]. However, since only a small fraction of T cells will have those changes, no impact on the efficacy of cell therapy would be expected. Importantly, T cells have been shown to be very resistant towards transformation [50] even after transduction with oncogene-expressing, mutagenic γ-retroviral vectors that efficiently transform haematopoietic stem cells [51]. In line, huge numbers of T cells transduced with this type of mutagenic γ-retroviral vectors have been infused in adoptive immunotherapy trials with chimaeric antigen receptor (CAR)- and TCR-transduced T cells. Moreover, in previous immunotherapy studies with TALEN- as well as CRISPR-Cas genome-edited T cells comparatively high frequencies (up to 4%) of translocations and deletions were found without impact on feasibility and safety [52, 53].

Altogether, mRNA electroporation of CCR5-Uco-hetTALEN was found to have a very-good safety profile. Even though the existence of ‘spontaneous’ off-targets might not be completely excluded, their likelihood is comparatively low, since dimerisation of the Fok1 nuclease is a sine qua non for DSB induction [54]. Indeed, the amplicon-NGS data for the TOP10 predicted off-targets are very promising and clearly indicated improved specificity of the obligatory heterodimeric new variant as compared to the previous CCR5-Uco-TALEN. Nevertheless, future studies will have to address the frequency of non-predicted off-targets with CCR5-Uco-hetTALEN in more detail using unbiased methods such as LAM-HTGTS [55, 56], Guide-seq [57] or BLISS [58]. As noted above, mRNA amounts need to be carefully adjusted in future clinical settings to minimise off-target cutting [20].

Interestingly, *CCR5* amplicon-NGS data from treated samples revealed an 18-bp deletion between the TALEN binding sites as the most frequent gene-editing event. This points to MMEJ as a major repair mechanism of DSBs used in edited primary T cells [59]. This notion is supported by our observation that the same 18-bp deletion was not present at the off-target *CCR2*, which lacks microhomology at this position. Evidently, an 18-bp deletion does not cause the indented frameshift mediating gene knockout, but instead results in the loss of six amino acids from the ICL1, only, with potentially limited impact on protein function, as was also suggested by protein modelling. This observation was surprising, since our previous data indicated good correlation between knockout frequencies measured on the DNA level and cell-surface expression of CCR5 [18]. Therefore, we studied the actual impact of the 6-aa deletion on CCR5 expression. To this end, we overexpressed WT CCR5 and mutated CCR5^Δ55-60^ in CCR5-deficient cells. Interestingly, we found that deletion of the six amino acids from ICL1 almost completely abrogated CCR5 export to the cell membrane. This is in accord with previous reports for the naturally occurring variants CCR5^R60S^ and CCR5^L55Q^ both also showing reduced cell-surface expression [60, 61]. Moreover, the leucine in the ICL1 (L61 in CCR5) is highly conserved among G-coupled receptors class A (84.7% in human). Mutation of this leucine in the α_2B_-adrenergic receptor (L48), for example, prevents endoplasmic reticulum export [62]. Together this data underlines the importance of the targeted region in ICL1 for efficient cell-surface expression of CCR5.

Our kinetics study revealed that gene-editing at on-target *CCR5* took place for 48–72 h post electroporation. Consequently, efficiency of *CCR5* gene-editing should not be determined earlier than 3 days post electroporation. Interestingly, *CCR2* off-target cutting by CCR5-Uco-hetTALEN was mainly confined to the first 24 h post electroporation supporting the notion that off-target editing preferentially occurs, when excess nuclease is available.

CNV between *CCR5* and reference gene *hEPOR* were the highest at 24 h post-electroporation probably reflecting extensive cutting at the target site at this time point. However, differences in copy numbers at later time points led us to conclude that some of the deletions induced by CCR5-Uco-hetTALEN span beyond the binding sites of PCR primers/probe used for GEF-dPCR and amplicon NGS, as was confirmed by the aberration-specific PCRs (s. above).

Contamination of cell products with introduced nuclear acids represents another potential safety issue. We found that CCR5-Uco-hetTALEN mRNA copy numbers decreased exponentially within 9 days after electroporation. On day 9, post-electroporation between 0 and 50 CCR5-Uco-hetTALEN mRNA copies were detected per 25.000 cells. Besides, we also measured residual donor plasmid in different CCR5-Uco-hetTALEN mRNA-treated samples by qPCR. At 120 h post-transfection, plasmid remains were only detected in one out of six experiments, and from 144 h after electroporation onwards, all samples were PCR negative.

Similar to the original homodimeric CCR5-Uco-TALEN, the new heterodimeric variant also facilitated high levels of biallelic editing (70% of edited cells). This translated into strong reduction of CCR5 cell-surface expression (22.0%-43.4%). Those results were supported by the infection-assay results, which also revealed reduction in HIV susceptibility of treated cells by similar rates.

We finally assessed functional properties of *CCR5*-edited T cells. To this end, we comparatively measured the proliferation capacity and cytokine secretion of *CCR5*-edited cells vs. non-edited control cells. Expectedly, both *CCR5*-edited and non-edited CD4^+^ T cells secreted high amounts of GM-CSF and INF-γ [63], while TNF-α and INF-α levels were low. No differences in expression kinetics for the cytokines investigated was found between edited and non-edited cells. Likewise, monitoring of T-cell proliferation after electroporation with or without TALEN revealed no differences. Together, this data supports that *CCR5* editing has no influence on T-cell fitness, as described by other groups, as well [41, 64].

CCR5-specific nucleases have been used in CD4^+^ T cells and CD34^+^ HSCs [65–69]. Modification of both cell types is associated with specific advantages and shortcomings. High-efficiency editing of HSCs might facilitate lifelong protection of all HIV-susceptible blood cells, including macrophages and microglia. On the downside, transplantation of HSCs nowadays requires myelotoxic conditioning associated with definitive short- and long-term risks. Moreover, an HSC-centred approach could result in the loss of all acquired adaptive CD4^+^ T-cell immunity, since existing T cells would not be protected. In contrast, protection of CD4^+^ T cells would conserve existing immunity, including potential HIV-specific clones, but other cells (e.g. macrophages) would potentially constitute a permanent HIV reservoir. Also, most edited T cells could be expected to be relatively short-lived, even though memory T cells exist for many decades, as was also shown for gene-modified T cells [70]. Moreover, T cells are largely resistant towards malignant transformation caused by genome modifications (s. above), a severe complication observed in several HSC-directed gene-therapy studies [71–73]. Thus, in an ideal scenario, such T-cell-directed strategy would provide the patient with a large proportion of long-lived HIV-resistant T cells maintaining his broad immune repertoire and thus preventing development of immune deficiency. In addition, protection of CD4^+^ T cells can be combined with other genetic modifications such as introduction of HIV- (or tumour-) specific CARs to facilitate anti-HIV or anti-cancer immunity [74].

Recently, Brec1, a Cre-recombinase-based designer enzyme that facilitates HIV provirus excision from infected cells’ genome [75], was suggested to be used for HIV gene therapy. Whereas CCR5-specific designer nucleases offer a ‘vaccine-like’ protection approach, Brec1 mediates ‘cure’ of already HIV-infected cells. Thus, the two approaches are highly complementary.

In summary, we successfully optimised our *CCR5*-targeting TALEN thus ensuring drastically reduced off-target activity. Notably, the majority of tested cells revealed biallelic *CCR5* gene-editing essential for efficient protection. We also confirmed that the targeted region of *CCR5* is particularly important for protein display at the cell surface. Besides the high *CCR5* gene-editing rates, we also showed a reduction of CCR5 cell-surface expression in TALEN-treated cells. These results were also confirmed by data from large-scale production of *CCR5*-edited cells using CCR5-Uco-hetTALEN in the CliniMACS Prodigy [20].

## Supplementary information

Supplementary information
