# Correction: Optimisation of a TALE nuclease targeting the HIV co-receptor CCR5 for clinical application

**DOI:** 10.1038/s41434-022-00373-y

**Published:** 2022-11-22

**Authors:** Lea Isabell Schwarze, Dawid Głów, Tanja Sonntag, Almut Uhde, Boris Fehse

**Affiliations:** 1 Department of Stem Cell Transplantation, Research Department Cell and Gene Therapy, University Medical Centre Hamburg-Eppendorf, Hamburg, Germany; 2 German Centre for Infection Research (DZIF), partner site Hamburg, Hamburg, Germany

**Keywords:** Infectious diseases, Transfection

Correction to: *Gene Therapy* 10.1038/s41434-021-00271-9, published online 11 June 2021

A wrong Supplementary file was originally published with this article due to a publisherʼs error; it has now been replaced with the correct file. The authors and the publisher apologize for any inconvenience.

## Supplementary information

Supplementary File

