# Supplementary material for: Optimisation of a TALE nuclease targeting the HIV co-receptor CCR5 for clinical application
Source: Gene Ther. 2021 Jun 11;28(9):588–601. doi: 10.1038/s41434-021-00271-9 (PMC8455333; doi:10.1038/s41434-021-00271-9)
Supplement: Supplementary file 1 — Supplementary information [file 41434_2021_271_MOESM1_ESM.docx]

**Optimisation of a TALE nuclease targeting the HIV coreceptor CCR5 for clinical application**

**Supplement figures Gene Therapy Paper**

**Table S1:** Search parameter used in web-based tool Paired Target Finder

|  | **Homodimeric TALEN** | **Heterodimeric TALEN** |
| --- | --- | --- |
| **Search Genome** | Homo sapiens (promoterome) | Homo sapiens (promoterome) |
| **RVD 1** | NK HD NG NK NK NG HD NI NG HD HD NG HD NI NG HD HD NG NN | NK HD NG NK NK NG HD NI NG HD HD NG HD NI NG HD HD NG NN |
| **RVD 2** | NI NN NI NG NN NG HD NI NK NG HD NI NG NK HD NG HD NG NG | NI NN NI NG NN NG HD NI NK NG HD NI NG NK HD NG HD NG NG |
| **Spacer length** | 15 – 30 | 10-30 |
| **Score cut off** | 4.0 | 4.0 |
| **Upstream base** | T or C | T or C |
| **Dimer types** | Both | Heterodimers only |
| **Scoring Matrix** | Doyle et al. | Doyle et al. |

**Table S2:** Search Parameter used in web-based tool PROGNOS

|  | **Homodimeric TALEN** | **Heterodimeric TALEN** |
| --- | --- | --- |
| **Nuclease Type** | TALEN | TALEN |
| **Sequence Entry** | Complementary Strand | Complementary Strand |
| **RVD left** | 01NK02HD03NG04NK05NK06NG07HD08NI09NG10HD11HD12NG13HD14NI15NG16HD17HD18NG19NN | 01NK02HD03NG04NK05NK06NG07HD08NI09NG10HD11HD12NG13HD14NI15NG16HD17HD18NG19NN |
| **RVD right** | 01NI02NN03NI04NG05NN06NG07HD08NI09NK10NG11HD12NI13NG14NK15HD16NG17HD18NG19NG | 01NI02NN03NI04NG05NN06NG07HD08NI09NK10NG11HD12NI13NG14NK15HD16NG17HD18NG19NG |
| **Max mismatches** | 7 | 7 |
| **Spacer length** | 10-30 | 10-30 |
| **Search for homodimeric sites** | Yes | No |
| **Genome to search** | Human (hg19) | Human (hg19) |
|  |  |  |

**Table S3:** Results from Paired Target Finder and PROGNOS for homodimeric CCR5-Uco-TALEN

| **Rank** | **Paired Target Finder - T only** | **Prognos RVD**  **-T only** | **Prognos RVD**  **- advanced** | **Prognos RVD**  **- all** | **Paired Target Finder - all** |  |
| --- | --- | --- | --- | --- | --- | --- |
| 1 | CCR5 | CCR5 | CCR5 | CCR5 | CCR5 |  |
| 2 | CCR2 | CCR2 | CCR5 | CCR2 | CCR2 |  |
| 3 | RAPGEF2 | RAPGEF2 | CCR2 | LDOC1 | RAPGEF2 |  |
| 4 | MUC16 | GPC5 | LDOC1 | SPANXA2-OT1 | LARGE |  |
| 5 | GPC5 | VWA8 | SPANXA2-OT1 | RAPGEF2 | MUC16 |  |
| 6 | VWA8 | BRS3 | RBMY1A3P | RBMY1A3P | KIRREL |  |
| 7 | BRS3 | LINC00534 | RAPGEF2 | TTTY13 | EPM2A |  |
| 8 | ARHGAP6 | TMTC2 | UBXN10 | LRRC7 | IQSEC2 |  |
| 9 | SEMA3C | SEMA3C | LRRIQ3 | RTN1 | GPC5 |  |
| 10 | IFNE | ARHGAP6 | TTTY13 | KIRELL | VWA8 |  |
|  |  |  |  |  |  |  |

**Table S4:** Results from Paired Target Finder and PROGNOS for heterodimeric CCR5-Uco-hetTALEN

| **Rank** | **Paired target finder** | **Prognos – T only** | **Prognos RVD** | **Prognos** |
| --- | --- | --- | --- | --- |
| 1 | CCR5 | CCR5 | CCR5 | CCR5 |
| 2 | CCR2 | CCR2 | CCR2 | CCR2 |
| 3 | CACNA1B | MAT2B | HS6ST3 | MAT2B |
| 4 | ASIC-202 | UBXN10 | LRRIQ3 | UBXN10 |
| 5 | SAMD12 | LRRIQ3 | ADCY2 | LRRIQ3 |
| 6 |  | ADCY2 | CSNK1G2 | SAMD12 |
| 7 |  | SAMD12 | EPHA7 | GLP1R |
| 8 |  | MTM1 | MTM1 | LOC286114 |
| 9 |  | CLVS1 | AGBL3 | LPHN2 |
| 10 |  | GLP1R | FAM41AY1 | KLHL4 |
| 11 |  | LOC286114 | DPP10 | PGC |
| 12 |  | EIF3IP1 | EIF3IP1 | NEK11 |
| 13 |  | LPHN2 | TMTC2 | LOC100124692 |
| 14 |  | KLHL4 | CXCR6 | ARHGAP10 |
| 15 |  | AACS | DAOA-AS1 | FMN1 |

**Table S5:** Selected loci of homodimeric CCR5-Uco-TALEN for off-target analysis by amplicon NGS

| (closest) gene | mismatches | chromosome | loci | TALEN 1 | TALEN 2 | Binding arm |
| --- | --- | --- | --- | --- | --- | --- |
| CCR5 | 0/0 | 3 | Exon | TGCTGGTCATCCTCATCCTG | TTCTCGTACTGACTGTAGAT | L+R |
| CCR2 | 3/3 | 3 | Exon | TGCTGGTCGTCCTCATCTTA | TTCACGAACTGACTGTAAAT | L+R |
| MUC16 | 5/5 | 19 | Exon | TGCATGTCCTCCTCATACTT | CTTCAACTCCCACTTGTCGT | L |
| VWA8 | 7/4 | 13 | Intron | TAAATGTCAGCTCTGCACAC | ATCTCGTAATGACCGTAGGT | R |
| KIRREL | 7/4 | 11 | Intron | CAGTTCTCAGCAATGTACTT | ATCGTGTACTGACTGTAGAC | R |
| GPC5 | 6/6 | 13 | Intron | TAAACATCAGTCTAGATCTT | AACCTGTACTAACTGTAAAT | R |
| IQSEC2 | 5/7 | X | Intron | CACATGTCTGTCCTGCTTTT | GTTTCGTACTGTCGATAAAC | R |
| BRS3 | 5/5 | X | Intergenic | TAGCTGTCAGGCATGTGCAT | TTCCCGTAAAGACGGAAGAT | R |
| RAPGEF2 | 5/6 | 4 | Intergenic | TAAAAATCAGTTATGCTCAT | TACTCGTCTTGACTAAAAAT | R |
| LDOC1 | 3/6 | X | Intergenic | CAAATGTCAGTCATGCACTT | TTCTGGTACTAACTAATGTT | R |
| SEMA3C | 5/5 | 7 | Intergenic | TAGAACTCAGTGCTGCTCTG | TTGGTGTCAGCCAAGCTCTG | R |

**Table S6:** Selected loci of heterodimeric CCR5-Uco-hetTALEN for off-target analysis by amplicon NGS

| (closest) gene | mismatches | chromosome | spacer length | loci | TALEN 1 | TALEN 2 |
| --- | --- | --- | --- | --- | --- | --- |
| CCR5 | 0/0 | 3 | 18 | exon | TGCTGGTCATCCTCATCCTG | AAGAGCATGACTGACATCTA |
| CCR2 | 3/3 | 3 | 18 | exon | TGCTGGTCGTCCTCATCTTA | AAGTGCTTGACTGACATTTA |
| CXCR6 | 7/5 | 3 | 12 | exon | TGCTGGTCATATCCATCTTC | CAGAGCCTGACGGATGTGTT |
| GLP1R | 5/5 | 6 | 24 | exon | TTCGGGTCATCTGCATCGTG | ATGTGCAAGACAGACATCAA |
| CACNA1B | 7/6 | 9 | 28 | intron | CAGCCTTAAGACATGCTCCT | CCCTGGCCATCCTCCTGCTC |
| ASIC-202 | 5/8 | 17 | 24 | intron | TTCTTGTCATCATCATCATA | TCAATGCCAGACCAGATCTC |
| SAMD12 | 6/4 | 8 | 22 | intron | TGTAAGTCAGACATTCTCAT | TGTTAGCCTTCCTCATCCTG |
| ADCY2 | 5/4 | 5 | 15 | intron | TGCGGGTGAGCCTCATCCAT | AAGAGAAAGACTGAAATCTC |
| PGC | 7/4 | 6 | 17 | promotor | TTGTTGTGGGCCCCATCCTG | AACAGCATTCCTGGCATCTA |
| MAT2B | 7/3 | 5 | 19 | intergenic | TATCTGGAGACCATGCTCTT | CAGGATGGAGGTGACCAGCA |
| UBXN10 | 7/3 | 1 | 24 | intergenic | TGGAGGTCAGACATGCCTCA | CAGGCCGAGGGTGACCAGCA |

**Table S7:** Samples used for amplicon NGS

| **Sample number** | **donor** | **Cell type** | **Cytokine** | **TALEN type** | **TALEN arms used** | **mRNA amount** | **mRNA production** |
| --- | --- | --- | --- | --- | --- | --- | --- |
| 1 | B | PBMCs | IL-2 | homodimeric | L | 10 µg | own lab |
| 2 | E |  |  |  | L | 10 µg | own lab |
| 3 | A |  |  |  | L+R | 10 µg | own lab |
| 4 | A |  |  |  | L+R | 20 µg | own lab |
| 5 | B |  |  |  | L+R | 10 µg | own lab |
| 6 | B |  |  |  | L+R | 10 µg | own lab |
| 7 | C |  |  |  | L+R | 10 µg | own lab |
| 8 | D |  |  |  | L+R | 10 µg | own lab |
| 9 | E |  |  |  | L+R | 10 µg | own lab |
| 10 | E |  |  |  | L+R | 10 µg | own lab |
| 11 | F | CD4^+^ | IL-2 | heterodimeric | Non | - | - |
| 12 | G | PBMCs | IL-2 |  | Non | - | - |
| 13 | H | PBMCs | IL-2 |  | L+R | 10 µg | own lab |
| 14 | I | PBMCs | IL-2 |  | L+R | 5 µg | own lab |
| 15 | G | PBMCs | IL-2 |  | L+R | 20 µg | own lab |
| 16 | F | CD4^+^ | IL-15 + IL-7 |  | L+R | 5 µg | BioNTech |
| 17 | F | CD4^+^ | IL-15 + IL-7 |  | L+R | 7.5 µg | BioNTech |
| 18 | F | CD4^+^ | IL-15 + IL-7 |  | L+R | 10 µg | BioNTech |
| 19 | J | CD4^+^ | IL-2 |  | L+R | 10 µg | own lab |

**Table S8:** Sequence of FokI variants

| **FokI Type** | **Sequence** |
| --- | --- |
| WT | CAACTCGTGAAGAGTGAACTTGAGGAGAAGAAGTCCGAGCTGCGGCACAAGCTGAAATACGTGCCCCACGAGTACATCGAGCTGATCGAGATCGCCAGAAACAGCACCCAGGACAGAATCCTGGAAATGAAGGTCATGGAGTTCTTTATGAAGGTGTACGGCTACCGGGGCAAGCACCTCGGAGGATCACGAAAGCCTGACGGCGCCATCTATACCGTGGGCAGCCCTATTGATTACGGCGTGATCGTGGATACCAAGGCCTACAGCGGCGGCTACAATCTGCCTATCGGGCAGGCCGACGAGATGCAGAGATACGTGGAAGAAAACCAGACACGGAACAAGCACATCAACCCCAACGAGTGGTGGAAAGTGTACCCCAGCAGCGTGACCGAGTTCAAGTTCCTGTTCGTGTCCGGCCACTTCAAGGGCAACTACAAGGCCCAGCTGACCCGGCTGAACCACATCACAAATTGCAACGGCGCTGTGCTGTCTGTGGAAGAACTGCTGATCGGCGGCGAGATGATCAAGGCCGGAACACTGACACTGGAAGAAGTGCGGCGGAAGTTCAACAACGGCGAGATCAACTTCCGGTCCTGA |
| DAD | CAACTCGTGAAGAGTGAACTTGAGGAGAAGAAGTCCGAGCTGCGGCACAAGCTGAAATACGTGCCCCACGAGTACATCGAGCTGATCGAGATCGCCAGAAACAGCACCCAGGACAGAATCCTGGAAATGAAGGTCATGGAGTTCTTTATGAAGGTGTACGGCTACCGGGGCAAGCACCTCGGAGGATCACGAAAGCCTGACGGCGCCATCTATACCGTGGGCAGCCCTATTGATTACGGCGTGATCGTGGATACCAAGGCCTACAGCGGCGGCTACAATCTGCCTATCGGGCAGGCCGACGAGATGCAGGACTACGTGGAAGAAAACCAGACCAGAGACAAGCACGCCAATCCTAACGAGTGGTGGAAAGTGTACCCCAGCAGCGTGACCGAGTTCAAGTTCCTGTTCGTGTCCGGCCACTTCAAGGGCAACTACAAGGCCCAGCTGACCCGGCTGAACCACATCACAAATTGCAACGGCGCTGTGCTGTCTGTGGAAGAACTGCTGATCGGCGGCGAGATGATCAAGGCCGGAACACTGACACTGGAAGAAGTGCGGCGGAAGTTCAACAACGGCGAGATCAACTTCCGGTCCTGA |
| ELD | CAACTCGTGAAGAGTGAACTTGAGGAGAAGAAGTCCGAGCTGCGGCACAAGCTGAAATACGTGCCCCACGAGTACATCGAGCTGATCGAGATCGCCAGAAACAGCACCCAGGACAGAATCCTGGAAATGAAGGTCATGGAGTTCTTTATGAAGGTGTACGGCTACCGGGGCAAGCACCTCGGAGGATCACGAAAGCCTGACGGCGCCATCTATACCGTGGGCAGCCCTATTGATTACGGCGTGATCGTGGATACCAAGGCCTACAGCGGCGGCTACAATCTGCCTATCGGGCAGGCCGACGAGATGGAACGCTACGTGGAAGAAAACCAGACCAGGGACAAGCACCTGAATCCTAACGAGTGGTGGAAAGTGTACCCCAGCAGCGTGACCGAGTTCAAGTTCCTGTTCGTGTCCGGCCACTTCAAGGGCAACTACAAGGCCCAGCTGACCCGGCTGAACCACATCACAAATTGCAACGGCGCTGTGCTGTCTGTGGAAGAACTGCTGATCGGCGGCGAGATGATCAAGGCCGGAACACTGACACTGGAAGAAGTGCGGCGGAAGTTCAACAACGGCGAGATCAACTTCCGGTCCTGA |
| KKR | CAACTCGTGAAGAGTGAACTTGAGGAGAAGAAGTCCGAGCTGCGGCACAAGCTGAAATACGTGCCCCACGAGTACATCGAGCTGATCGAGATCGCCAGAAACAGCACCCAGGACAGAATCCTGGAAATGAAGGTCATGGAGTTCTTTATGAAGGTGTACGGCTACCGGGGCAAGCACCTCGGAGGATCACGAAAGCCTGACGGCGCCATCTATACCGTGGGCAGCCCTATTGATTACGGCGTGATCGTGGATACCAAGGCCTACAGCGGCGGCTACAATCTGCCTATCGGGCAGGCCGACGAGATGCAGAGATACGTGAAAGAGAACCAGACACGGAACAAGCACATCAACCCCAACGAGTGGTGGAAAGTGTACCCCAGCAGCGTGACCGAGTTCAAGTTCCTGTTCGTGTCCGGCCACTTCAAGGGCAACTACAAGGCCCAGCTGACCCGGCTGAACAGGAAGACAAATTGCAACGGCGCTGTGCTGAGCGTGGAAGAACTGCTGATCGGCGGCGAGATGATCAAGGCCGGAACACTGACACTGGAAGAAGTGCGGCGGAAGTTCAACAACGGCGAGATCAACTTCCGGTCCTGA |
| RVR | CAACTCGTGAAGAGTGAACTTGAGGAGAAGAAGTCCGAGCTGCGGCACAAGCTGAAATACGTGCCCCACGAGTACATCGAGCTGATCGAGATCGCCAGAAACAGCACCCAGGACAGAATCCTGGAAATGAAGGTCATGGAGTTCTTTATGAAGGTGTACGGCTACCGGGGCAAGCACCTCGGAGGATCACGAAAGCCTGACGGCGCCATCTATACCGTGGGCAGCCCTATTGATTACGGCGTGATCGTGGATACCAAGGCCTACAGCGGCGGCTACAATCTGCCTATCGGGCAGGCCAGAGAGATGCAGAGATACGTCGAGGAAAACCAGACACGGAACAAGCACATCAACCCCAACGAGTGGTGGAAAGTGTACCCCAGCAGCGTGACCGAGTTCAAGTTCCTGTTCGTGTCCGGCCACTTCAAGGGCAACTACAAGGCCCAGCTGACCCGGCTGAACAGAGTGACAAATTGCAACGGCGCTGTGCTGAGCGTGGAAGAACTGCTGATCGGCGGCGAGATGATCAAGGCCGGAACACTGACACTGGAAGAAGTGCGGCGGAAGTTCAACAACGGCGAGATCAACTTCCGGTCCTGA |

**Table S9:** Primers and probes used.

| **Name** | **Sequence** | **Purification** | **Modification** |
| --- | --- | --- | --- |
| CCR5fw | CTGCCTCCGCTCTACTCACT | HPSF |  |
| CCR5rv | CCCAGAAGGGGACAGTAAGA | HPSF |  |
| CCR5ref | CTTTGGTTTTGTGGGCAACATGC | HPLC | 5‘ HEX 3‘-BHQ1 |
| CCR5mut | CTGCAAAAGGCTGAAGAGCATGAC | HPLC | 5‘ FAM 3‘-BHQ1 |
| CCR2fw | CAAATTGGGGCCCAACTC | HPSF |  |
| CCR2rv | GCCCACAATGGGAGAGTAATA | HPSF |  |
| CCR2mut | CTGCAAAAAGCTGAAGTGCTTGACTG | HPLC | 5‘ FAM 3‘-BHQ1 |
| hEPORfw | GCTGCCAGCTTTGAGTACACTA | HPSF |  |
| hEPORrv | GAGATGCCAGAGTCAGATACCACAA | HPSF |  |
| hEPORref | TTCTGAGGCGCCACTTTTGCAAGACC | HPLC | 5‘ FAM 3‘-BHQ1 |
| Inv1fw | GAAGCAAATCGCAGCCC | HPSF |  |
| Int1rv | TATGCACAGGGTGGAACAAG | HPSF |  |
| hetTALENfw | CAAGCACCTCGGAGGATCAC | Salt-free |  |
| hetTALENrv | ATAGGCAGATTGTAGCCGCC | Salt-free |  |
| IPO8fw | GACCGTTCCTCCTGAGACTC | Salt-free |  |
| IPO8rv | TCCTGGGCTTCCATATCGTTC | Salt-free |  |
| Kanfw | GAGCTGATACCGCTCGCC | Salt-free |  |
| Kanrv | CATCCGTCAGGGGCCTTC | Salt-free |  |
| nesPCRfw | CATTCATGGAGGGCAACTAAATAC | HPSF |  |
| nesPCRrv | CGATTGTCAGGAGGATGATG | HPSF |  |
| HRMfw | GGTTTTGTGGGCAACATGCTGG | HPSF |  |
| HRMrv | GGTCAGAGATGGCCAGGTTG | HPSF |  |
| Rccr5del6fw | ATGACTGACATCTACCTGCTCAACCTGGC | Salt-free |  |
| Fccr5del6rv | GTTTATCAGGATGAGGATGACCAGCATGTTGC | Salt-free |  |


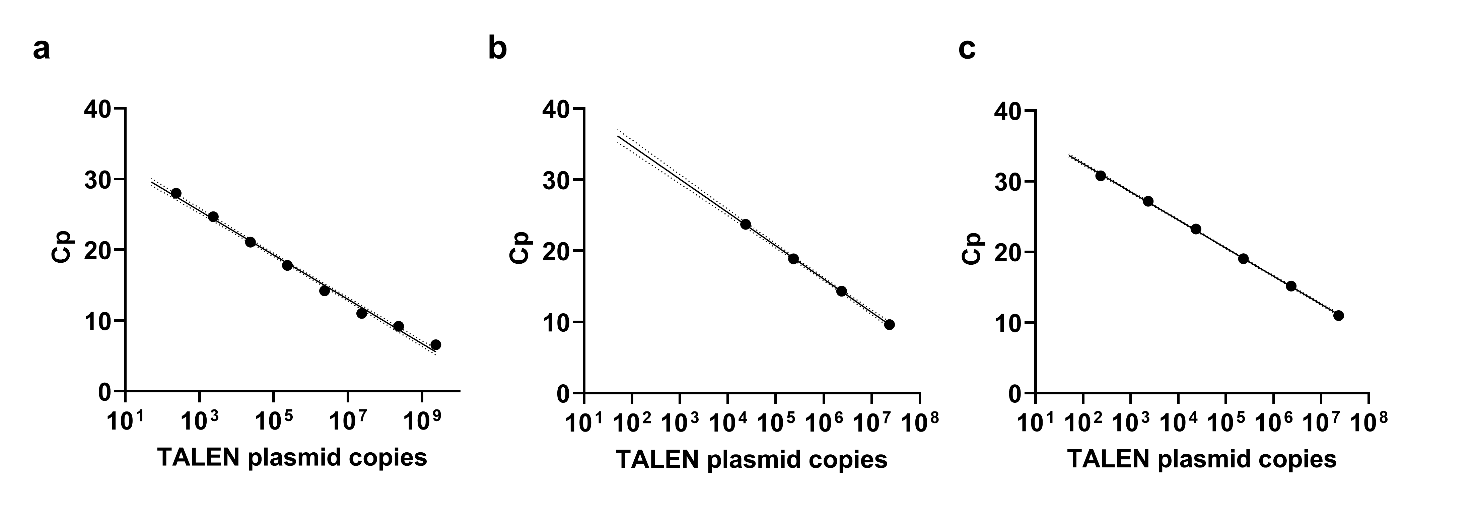


**Figure S1:** Standard curves for dilution of CCR5-Uco-hetTALEN plasmid for detection of CCR5-Uco-hetTALEN mRNA and plasmid. CCR5-Uco-hetTALEN plasmids L+R were diluted resulting in indicated copy numbers. C_q_ values were determined by qPCR in triplicates. A line (semilog) was fitted to data points by nonlinear regression with least square regression using GraphPad Prism 8.4.3. Dotted lines indicated predictions bands with a confidence interval of 95%. (a) qPCR for detection of CCR5-Uco-hetTALEN with primers hetTALENfw and hetTALENrv. R^2^ value = 0.9921. (b) qPCR for detection of CCR5-Uco-hetTALEN plasmid in RNA isolates with primers Kanfw and Kanrv. R^2^ value = 0.9959. (b) qPCR for detection of CCR5-Uco-hetTALEN plasmid in RNA isolates with primers Kanfw and Kanrv. R^2^ value = 0.9993.


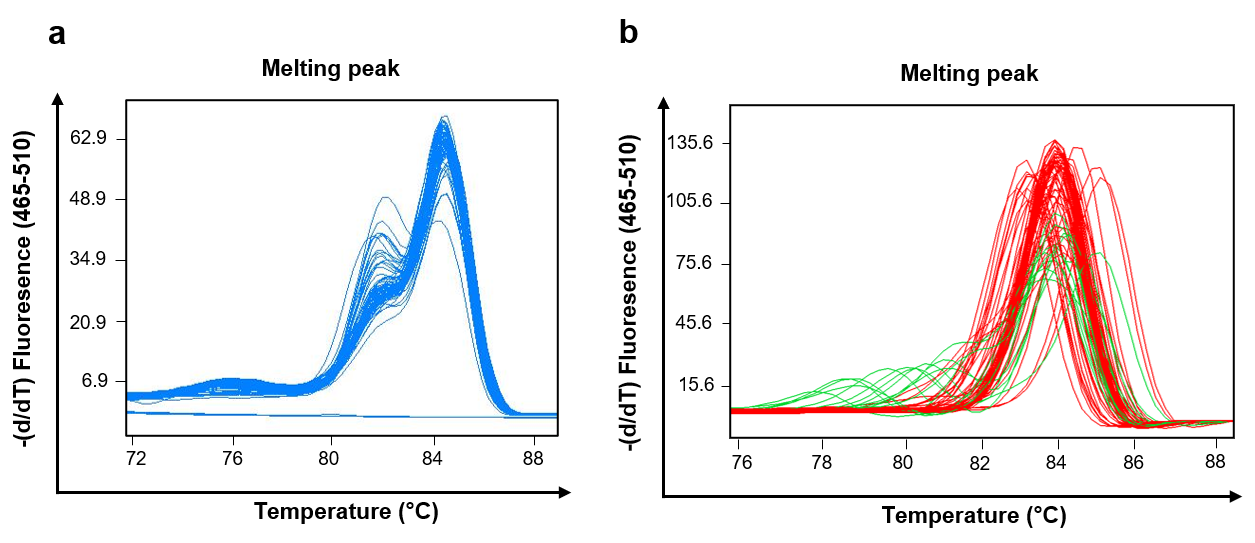


**Figure S2:** Development of a single-cell High-Resolution Melting Curve Analyses (scHRMCA) protocol for the detection of mono- and biallelic alleles. (a) scHRMCA results from primary human T cells of a heterozygous *CCR5*Δ32 donor. After first PCR diluted PCR product was used as a template for melting curve analysis as described in the method part using the following primer: fw CTTCATTACACCTGCAGCTCTCATT and rv CCCGAGTAGCAGATGACCATG. –(d/dT) Fluorescence plotted against temperature in °C shows monoallelic (32 bp deletion in one *CCR5* allele) deletion in all wells containing cells. Even though melting peak hight differed probably due to pipetting difference all cells could be called correctly as heterologous. (b) Example plot of scHRMCA results from primary human T cells treated with CCR5-Uco-TALEN. scHRMCA was performed as described in methods. Red lines show homologous melting profiles, while the green lines resemble heterologous melting curves as called by the LightCycler software. Correct calling of melting curve profiles was confirmed by Sanger sequencing in selected wells (data not shown). Consistence of results and correct calling was tested in three runs.


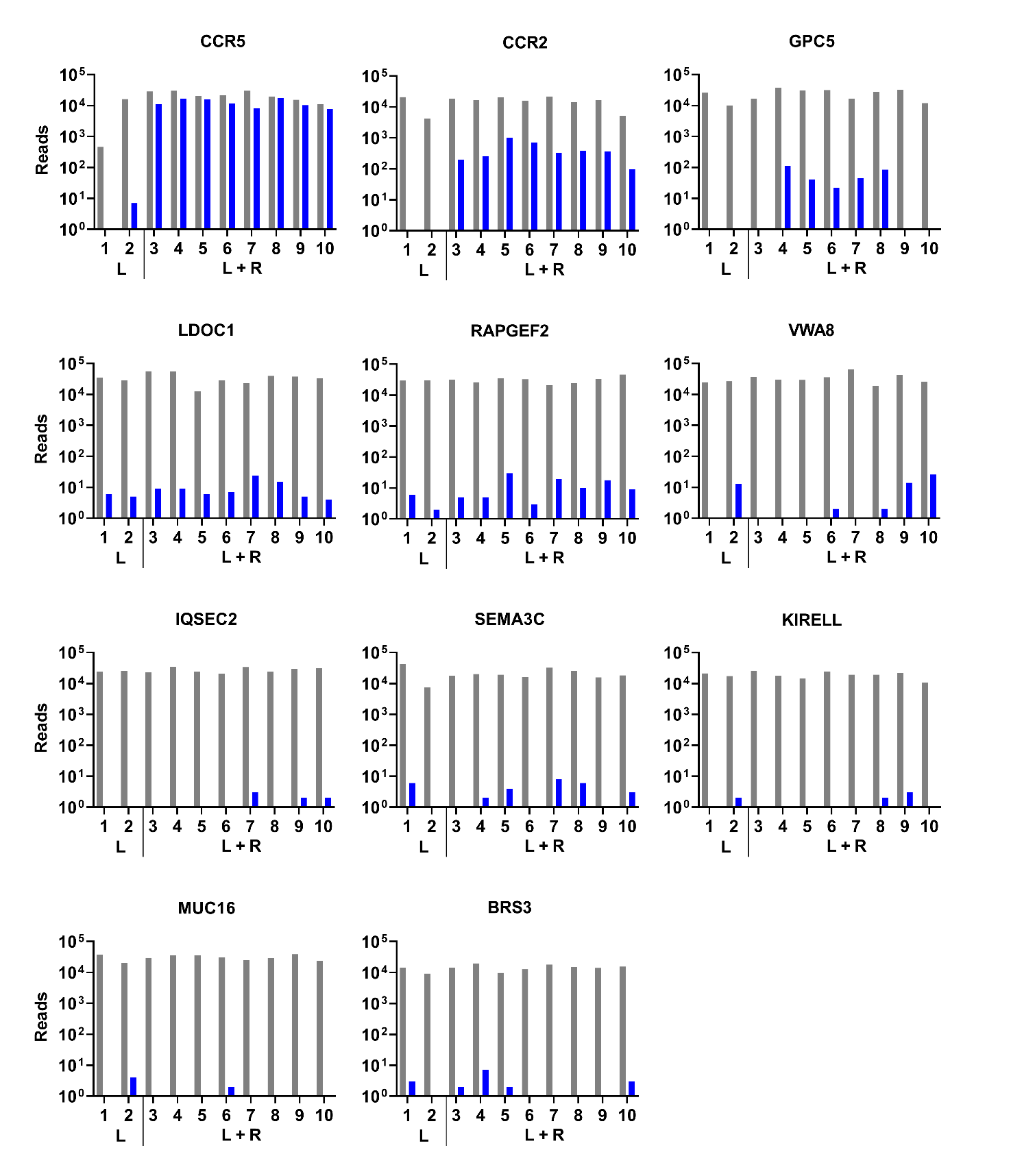


**Figure S3:** Next-generation amplicon sequencing data (read counts) from samples (n=8) treated with homodimeric CCR5-Uco-TALEN (TLN L+R) and samples treated with left CCR5-Uco-TALEN (TLN  L) only (n=2) for on-target CCR5 and 10 potential off-targets. Reads containing insertions or deletions (Indels) at the TALEN-binding sites were counted as Indel reads (right, blue bars), while all other reads were considered non-edited (left, grey bars).


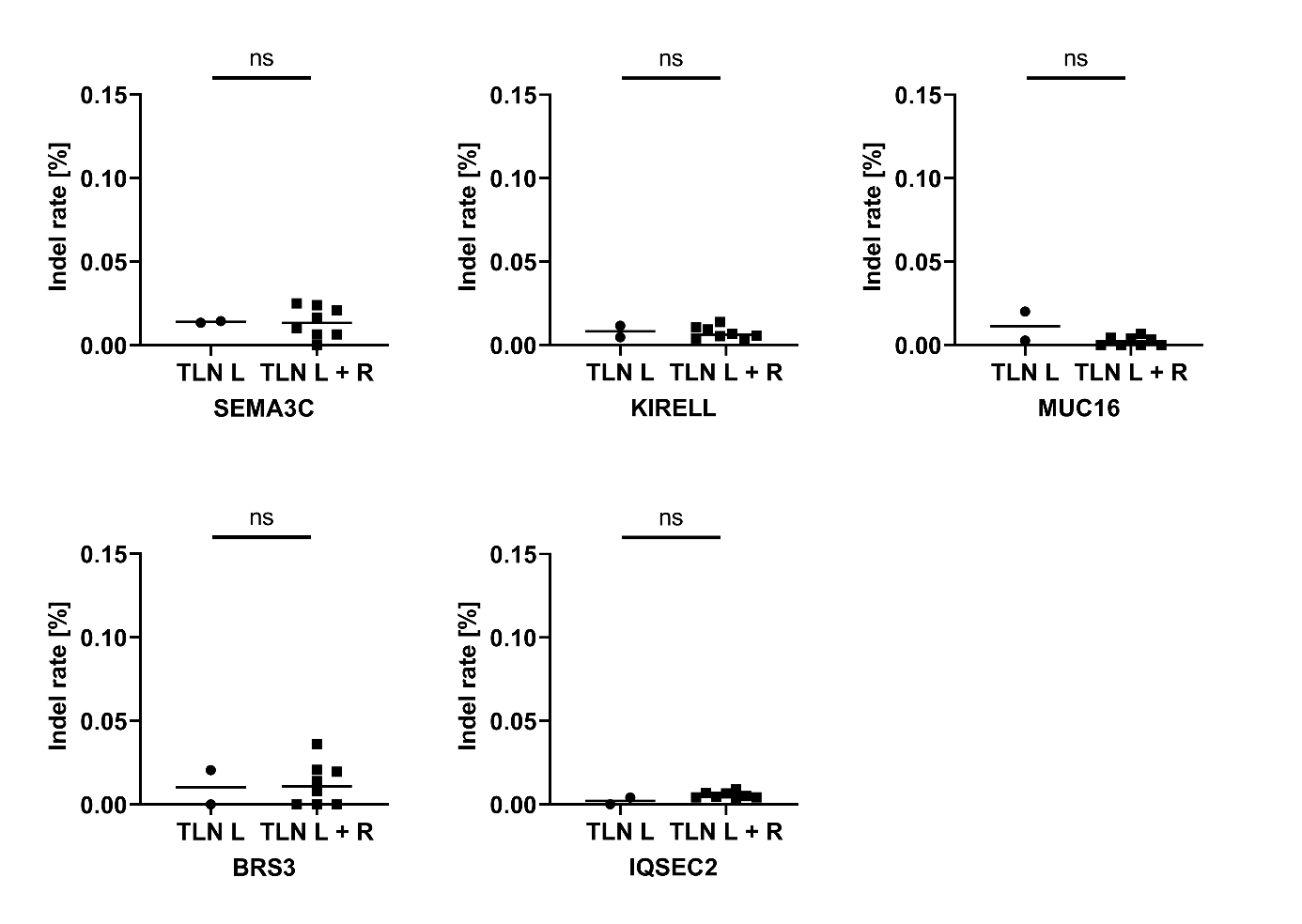


**Figure S4:** Next-generation amplicon sequencing data from samples (n=8) treated with homodimeric CCR5-Uco-TALEN (TLN L+R) and samples treated with left CCR5-Uco-TALEN (TLN L) only (n=2) for potential off-targets SEMA3C, KIRELL, MUC16, BRS3 and IQSEC2. Reads containing insertions or deletions (Indels) at the TALEN-binding sites were counted as Indel reads, while all other reads were considered non-edited. Indel rates were calculated using the ratio of reads containing Indels to all reads. Statistical analysis of Indel ratio was done using a one-tailed Welch’s t test with a confidence interval of 95%. P values: ns p > 0.12


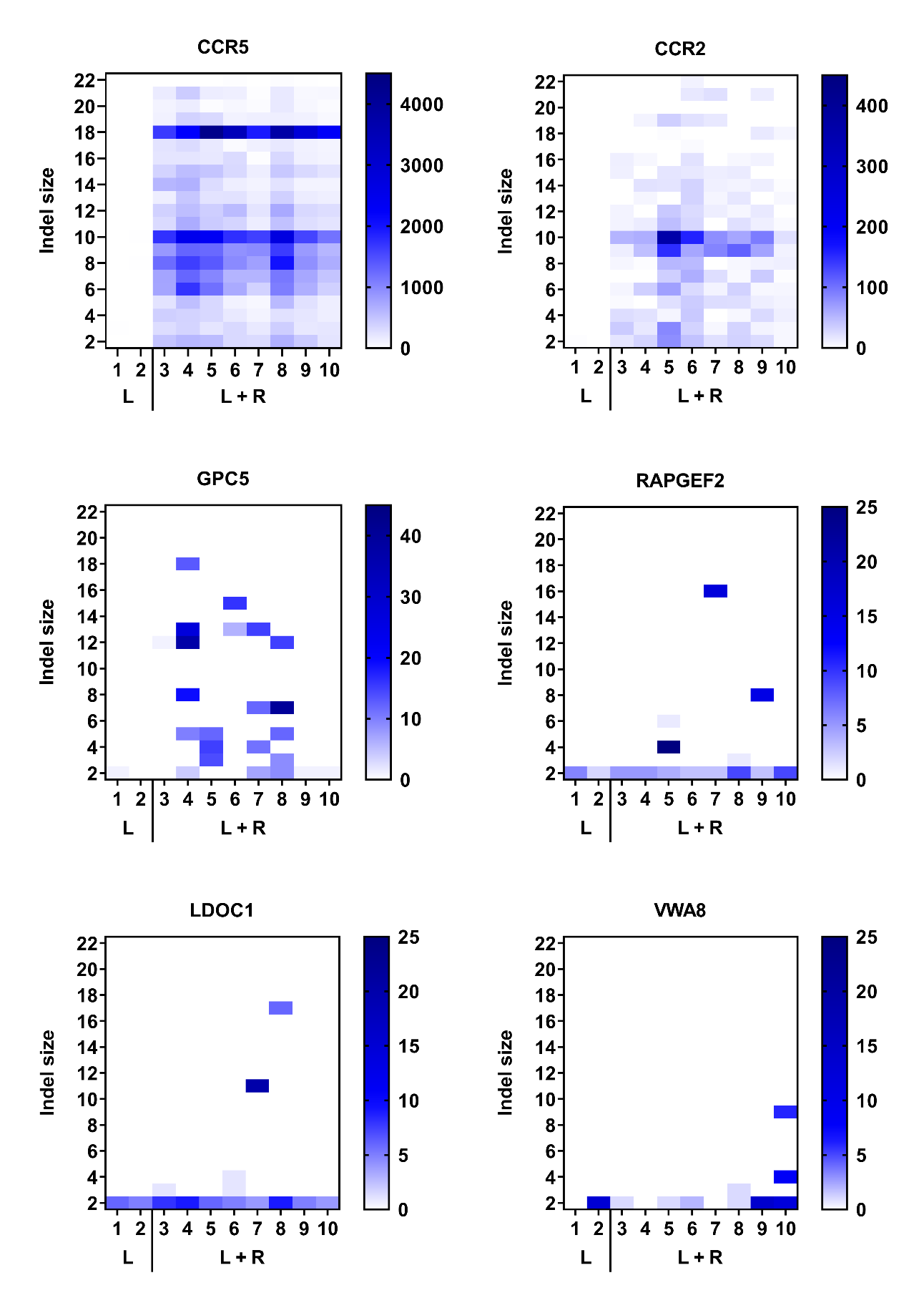


**Figure S5:** Next-generation amplicon sequencing data from samples (n=8) treated with homodimeric CCR5-Uco-TALEN L+R and samples treated with left CCR5-Uco-TALEN only (n=2) for on-target CCR5 and potential off-targets CCR2, GPC5, RAPGEF2, LDOC1 and VWA8. Reads containing insertions or deletions (Indels) at the TALEN-binding sites were counted as Indel reads. Indel read numbers are plotted based on Indel sizes (deletions or insertions of 2-22 base pairs).


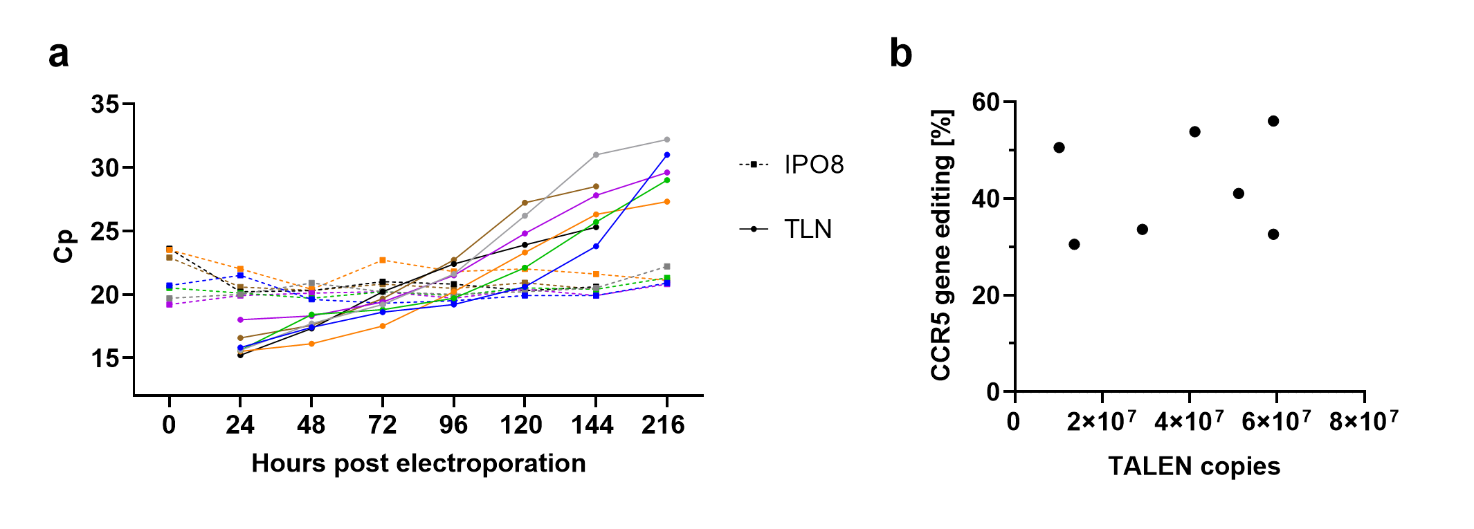


**Figure S6:** mRNA kinetic experiment. Primary T cells from 7 different donors were treated with 3 µg of CCR5-Uco-hetTALEN_poly(A) L+R mRNA per 2x10^6^ cells. Samples were taken before and every 24 h post electroporation. (a) C_p_ values for CCR5-Uco-hetTALEN mRNA (continuous line with dots) and Importin 8 (IPO8, dashed line with squares). Identical colours indicate the same experiment (b) CCR5-Uco-hetTALEN mRNA copy numbers at 24h post electroporation plotted against CCR5 gene editing rate at 144h post electroporation. Primary T cells from 7 different donors were treated with 3 µg of CCR5-Uco-hetTALEN_poly(A) L+R mRNA per 2x10^6^ cells. TALEN copy numbers are calculated based on the C_P_ values from a standard curve with defined TALEN plasmid copy numbers. CCR5 gene editing rates were determined by GEF-dPCR.


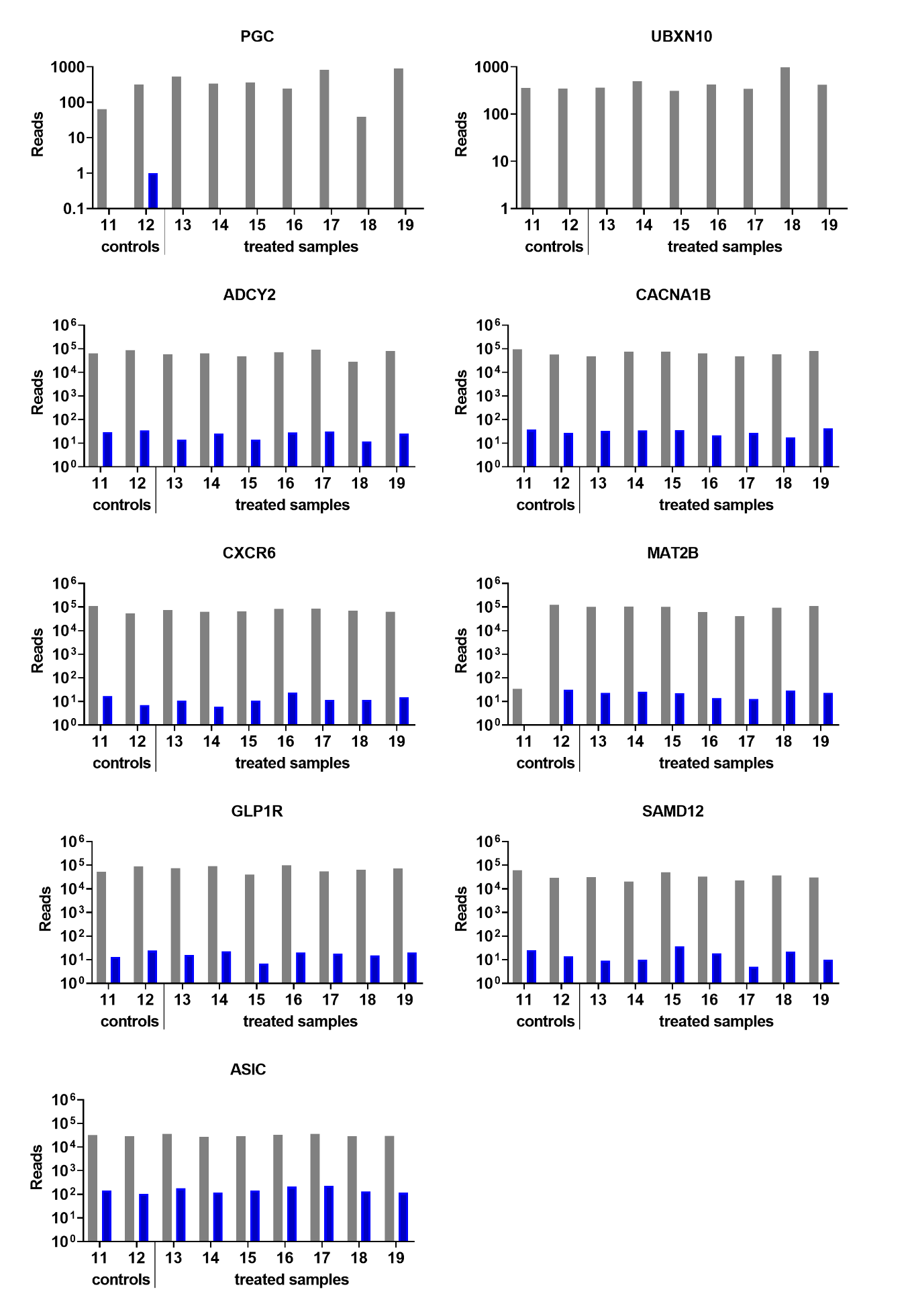


**Figure S7:** Next-generation amplicon sequencing data (read counts) from samples (n=9) treated with heterodimeric CCR5-Uco-hetTALEN (TLN L+R) and non-treated samples (controls) samples (n=2) for 9 potential off-targets PGC, UBXN10, ADYC2, CACNA1B, CXCR6, MAT2B, GLP1R, SAMD12, ASIC. Reads containing insertions or deletions (Indels) at the TALEN-binding sites were counted as Indel reads (right, blue bars), while all other reads were considered non-edited (left, grey bars).


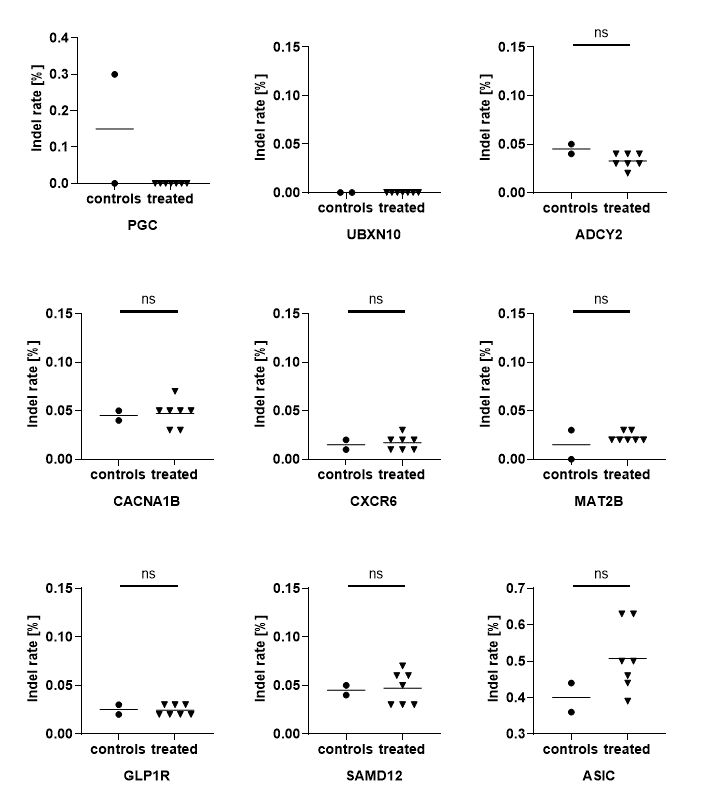


**Figure S8:** Next-generation amplicon sequencing data from samples (n=8) treated with heterodimeric CCR5-Uco-hetTALEN (TLN L+R) and non-treated samples (controls) samples (n=2) for 9 potential off-targets PGC, UBXN10, ADYC2, CACNA1B, CXCR6, MAT2B, GLP1R, SAMD12, ASIC. Reads containing insertions or deletions (Indels) at the TALEN-binding sites were counted as Indel reads, while all other reads were considered non-edited. Indel rates were calculated using the ratio of reads containing Indels to all reads. Statistical analysis of Indel ratio was done using a one-tailed Welch’s t test with a confidence interval of 95%. No statistical analysis was performed for PGC and UBXN10 due to low read numbers. P values: ns p > 0.12


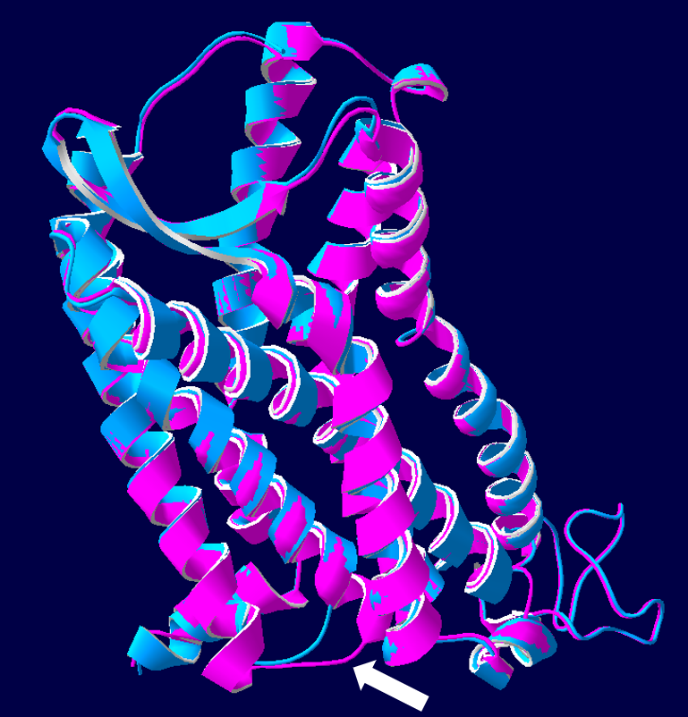


**Figure S9:** Superimposed protein model of wildtype CCR5 (pink) and CCR5^Δ55-60^ (blue) computed using DeepView/Swiss-PdbViewer (Version 4.1.0) from the Swiss Institute of Bioinformatic (http://www.expasy.org/spdbv/). Protein models for both CCR5 variants were calculated based on amino acid sequences with the web-based program for homology modelling of protein structures SWISS-MODEL from the University of Basel, Centre for Molecular Life Sciences (1-5).


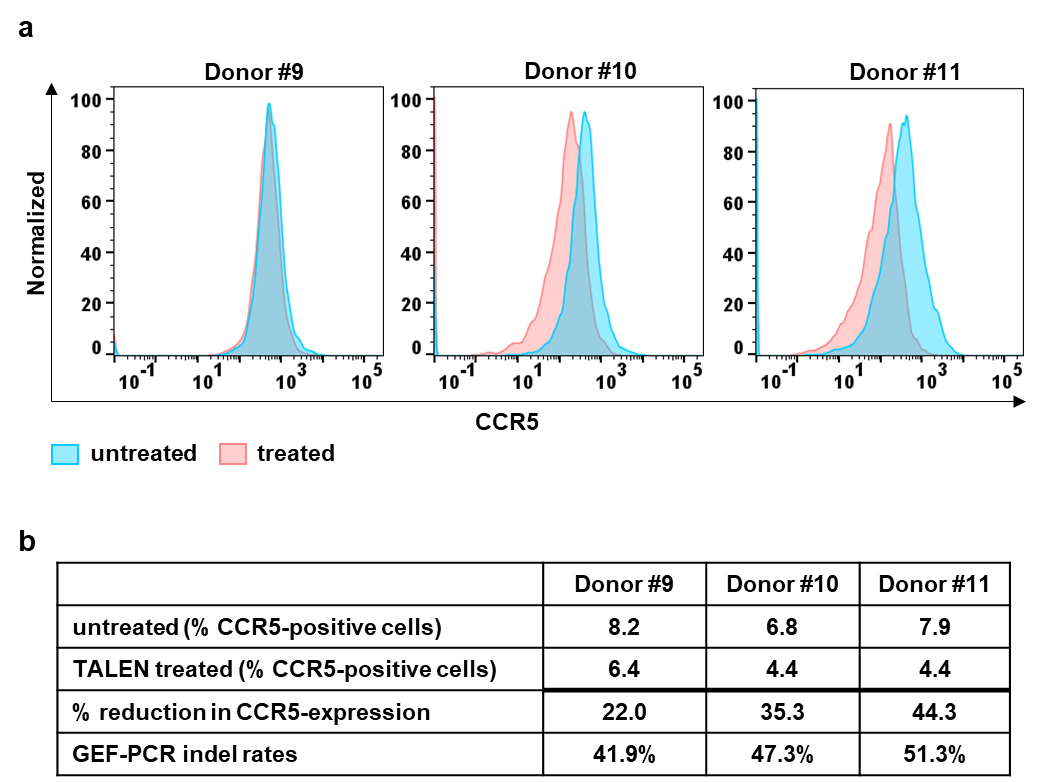


**Figure S10:** CCR5 surface expression of edited and non-edited CD4^+^ T cells. Primary human CD4^+^ T cells from three different donors were electroporated with 2.5 μg of CCR5-Uco-hetTALEN_polyA L+R mRNA per 1x10^6^ cells or without mRNA. 7 days post electroporation CCR5-edited (treated) and non-edited (untreated) cells were stained for viability and CCR5-expression. (a) Histograms showing CCR5-expression of untreated (blue) and treated (red) cells from three different donors (#9-#11). (b) Summarized data of the reduction in CCR5-expression and GEF-PCR results from the three tested donors. CCR5-expression was gated on viable cells in dot plots.


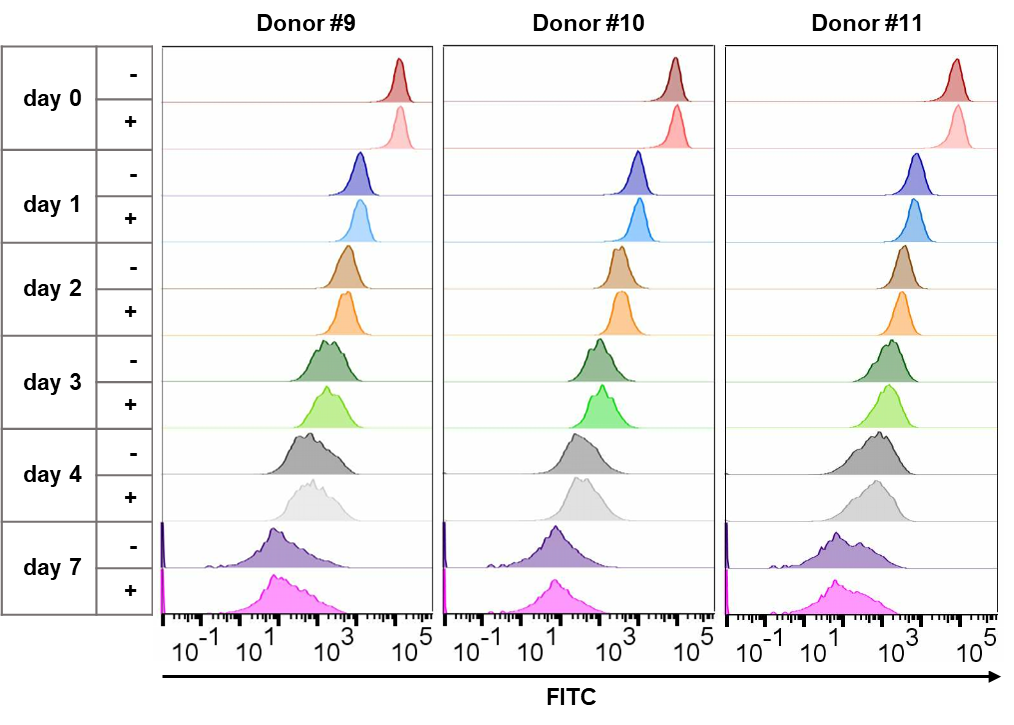


**Figure S11:** Proliferation capacity of CCR5-edited CD4+-T cells. Primary human CD4^+^ T cells from three different donors (donor #9-#11) were electroporated with 2.5 μg of CCR5-Uco-hetTALEN_polyA L+R mRNA per 1x10^6^ cells or without mRNA. GEF-dPCR results showed Indel rates for TALEN-treated cells of 37.2% for donor #9, of 46.4% for donor #10 and 42.2% for donor #11. Indel rates of non-edited cells from all donors were < 0.7%. Proliferation of cells was monitored after staining with CellTrace CSFE for 7 days by flow cytometry. Graph shows the measured fluorescence for CSFE (FITC channel) on each day of measurement for CCR5-edited (+) and non-edited (-) cells.

**Figure S12:** Cytokine secretion capacity of CCR5-edited CD4^+^-T cells. Primary human CD4^+^ T cells from three different donors were electroporated with 2.5 μg of CCR5-Uco-hetTALEN_polyA L+R mRNA per 1x10^6^ cells or without mRNA. Secreted cytokines (GM-CSF, IFN-α, IFN-γ, IL-2, IL-4, IL-5, IL 6, IL-9, IL-10, IL-12p70, IL-17A and TNF-α) were measured in the medium of non-edited (w/o TLN; CCR5 Indel rate of 0.6±0.2%) and CCR5-edited (+ TLN; CCR5 Indel rate 48.7±2.8%) cells on day 6 (d6) and day 12 (d12) post activation. Results show mean values from three different donors each measured in duplicates. Error bars show SD values. No significant difference was calculated in GraphPad Prism v.9.0.0 using a multiple unpaired t test.

**References**

1. Arnold K, Bordoli L, Kopp J, Schwede T. The SWISS-MODEL workspace: a web-based environment for protein structure homology modelling. Bioinformatics. 2006 Jan;22(2):195–201.

2. Biasini M, Bienert S, Waterhouse A, Arnold K, Studer G, Schmidt T, et al. SWISS-MODEL: Modelling protein tertiary and quaternary structure using evolutionary information. Nucleic Acids Res. 2014;42(W1):252–8.

3. Waterhouse A, Bertoni M, Bienert S, Studer G, Tauriello G, Gumienny R, et al. SWISS-MODEL: Homology modelling of protein structures and complexes. Nucleic Acids Res. 2018;46(W1):W296–303.

4. Guex N, Peitsch MC. SWISS-MODEL and the Swiss-Pdb Viewer: An environment for comparative protein modeling. Electrophoresis. 1997 Jan 1;18(15):2714–23.

5. Benkert P, Biasini M, Schwede T. Toward the estimation of the absolute quality of individual protein structure models. Bioinformatics. 2011;27(3):343–50.
